# Acid-cleavable thiomaleamic acid linker for homogeneous antibody–drug conjugation†
†Electronic supplementary information (ESI) available: ^1^H and ^13^C spectra for all novel compounds, and ES-MS spectra for all reactions with proteins described herein. See DOI: 10.1039/c3cc45220dClick here for additional data file.



**DOI:** 10.1039/c3cc45220d

**Published:** 2013-08-09

**Authors:** Lourdes Castañeda, Antoine Maruani, Felix F. Schumacher, Enrique Miranda, Vijay Chudasama, Kerry A. Chester, James R. Baker, Mark E. B. Smith, Stephen Caddick

**Affiliations:** a Department of Chemistry , University College London , 20 Gordon Street , London , WC1H 0AJ , UK . Email: m.e.b.smith@ucl.ac.uk ; Email: vpenterprise@ucl.ac.uk ; Tel: +44 (0)20 7679 7538; b UCL Cancer Institute , 72 Huntley Street , London , WC1E 6BT , UK

## Abstract

In this communication we describe a novel acid-cleavable linker strategy for antibody–drug conjugation. Functional disulfide bridging of the single interchain disulfide bond of a trastuzumab Fab fragment yields a homogeneous antibody–drug conjugate bearing a thiomaleamic acid linker. This linker is stable at physiological pH and temperature, but quantitatively cleaves at lysosomal pH to release the drug payload.

## Abstract 2

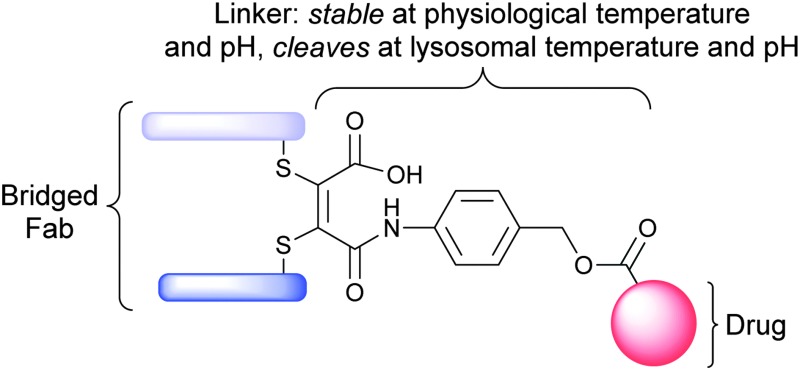
Homogeneous antibody–drug conjugation is affected using a novel thiomaleamic acid linker that is stable at physiological temperature and pH, but quantitatively cleaves at lysosomal pH to release the drug payload.

Therapeutic antibodies are large proteins that have been developed and optimised to specifically target discrete proteins and cells.^1^ Typically, the target will be a cell surface antigen that is either uniquely- or over-expressed in response to a pathological condition. Once bound to its target, the antibody stimulates the patient's immune system to attack and destroy the diseased cells.^1^ As of March 2012, 34 monoclonal antibodies (mAbs) had been approved for use in various disease indications, including cancer.^2^ However, despite their exquisite target selectivity, antibodies are often not efficacious enough to provide the desired therapeutic effect in isolation and are often prescribed in combination with a non-specific chemotherapy.^3^

Antibody–drug conjugates (ADCs) comprise an antibody that is armed with a highly potent, small molecule warhead using an appropriate conjugation/linker technology.^3,4^ They constitute a new class of targeted therapy that has shown considerable promise in the treatment of a variety of diseases, particularly cancer. For this indication, there are currently two FDA-approved ADCs and 30 ADCs in the clinic.^5^ For ADCs to deliver their full potential, the development of sophisticated conjugation/linker technologies to connect the potent warhead to the antibody is vital.^6^ An ideal linker must be stable in blood plasma, to avoid issues of premature warhead release and off-target toxicity, but cleave to release the warhead once the ADC has been internalised into its target cells and, commonly, trafficked to the lysosome. Consideration of how the linker is conjugated to the antibody is also critical. Conjugation to ADCs is currently typically achieved *via* either lysine modification or by functionalisation of partially-reduced interchain disulfide bonds.^6^ Lysine modification is sub-optimal as deleterious conjugation to lysine residues in the antigen binding region of the antibody can occur. Moreover, it generates heterogeneous ADC products, which have been shown to have an appreciably narrow therapeutic index relative to homogeneous ADCs, in pre-clinical studies.^7^ Cysteine modification, following interchain disulfide reduction, is also sub-optimal as it leads to the permanent loss of structural disulfide bonds that may reduce the stability of the ADC *in vivo*.

We report herein a new linker/conjugation strategy, based on thiomaleamic acid chemistry, with considerable potential in the ADC arena. The strategy allows for the generation of a homogeneous ADC product *via* functional, antibody interchain disulfide-bridging that displays retention of binding to its target antigen. In addition, the linker is stable at physiological temperature and pH but cleaves at mildly acidic, lysosomal pH, thus creating a mechanism for targeted-warhead release.

We have previously reported that activated maleimides can be used to functionally re-bridge disulfide bonds in an engineered antibody scFv fragment and peptide hormones to yield homogeneous products.^8^ We have also shown that partial hydrolysis of *N*-arylthiomaleimide bioconjugates can be achieved by judicious control of buffer pH.^8*b*^ The subsequent ring opening generates a thiomaleamic acid bioconjugate that is stable at both physiological conditions and millimolar concentrations of thiol for protracted periods. It has been previously reported that maleamic acids are labile in acidic pH, providing a mechanism for the controlled release of the amine linked through the amide functionality.^9^ We rationalised, therefore, that we could combine these different facets of our methodology to generate homogeneous ADCs that would selectively release a drug cargo at acidic, lysosomal pH.

Our study began with an evaluation of the hydrolysis behaviour of dithiomaleimide **2**. This construct was prepared by reaction of dibromomaleic anhydride with aniline to afford dibromomaleimide **1**, followed by subsequent treatment with ethanethiol. With dithiomaleimide **2** in-hand, hydrolysis was affected using LiOH·H_2_O to generate unsymmetrical compound **3**. Pleasingly, acidification to pH 4 of the reaction mixture resulted in cleavage of the amide bond, release of aniline and formation of anhydride **4** in quantitative yield (Scheme 1).

**Scheme 1:**
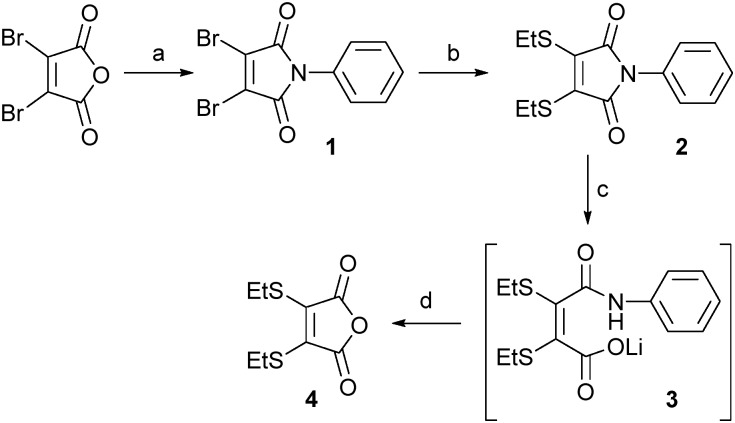
Small molecule partial hydrolysis/cleavage study: (a) PhNH_2_, AcOH, rt to 130 °C, 57%; (b) EtSH, NEt_3_, CH_2_Cl_2_, 93%; (c) LiOH·H_2_O, CD_3_OD : D_2_O (1 : 1); (d) 2 M HCl to pH 4, >99% (over two steps).

Encouraged by the successful hydrolysis and cleavage of dithiomaleimide **2**, we progressed to design a linker that combined facets of the acid-promoted cleavage of dithiomaleamic acids with the known self-immolative *p*-aminobenzyloxycarbonyl (PABC) spacer.^10^ In the first instance, we focused on a study of model conjugate **7** (Scheme 2). To generate this construct, 2,3-dibromomaleimide was treated with methylchloroformate to generate activated maleimide **5**,^11^ which on reaction with 4-aminobenzyl alcohol gave **6** in quantitative yield. Subsequent treatment with ethanethiol, followed by reaction with phenylisocyanate afforded the desired model conjugate, **7**.

**Scheme 2:**
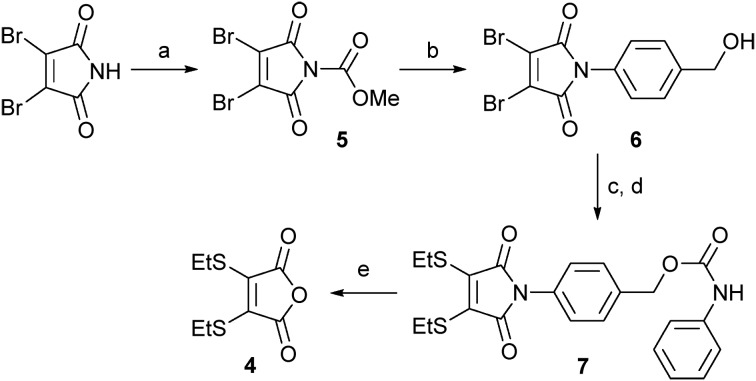
Synthesis and cleavage study of thiomaleamate linker **7**: (a) MeOCOCl, NMM, THF, 97%; (b) PABA, CH_2_Cl_2_, 99%; (c) EtSH, NEt_3_, CH_2_Cl_2_, 86%; (d) PhNCO, NEt_3_, CH_2_Cl_2_, 62%; (e) LiOH·H_2_O, CD_3_OD : D_2_O (1 : 1), then 2 M HCl to pH 4, >99%.

The partial alkaline hydrolysis of **7** using LiOH·H_2_O provided an unsymmetrical compound, analogous to that observed for compound **2**. Gratifyingly, adjustment to pH 4 resulted in cleavage of the linker, affording anhydride **4** in quantitative yield and the released aniline in 95% yield.

Following our successful proof of concept studies for the combined dithiomaleimide–PABC linker strategy on a small molecule model system, we focused our attention on developing a linker-drug conjugate that would be suitable for conjugation to an antibody fragment. For the purposes of this work we selected the anti-cancer drug doxorubicin (DOX) as a suitable warhead.^10,12^ We have previously reported the utility of dithiophenolmaleimides for achieving the functional re-bridging of disulfide bonds in peptides and proteins.^8a,c^ We therefore selected linker-drug construct **10** as an ideal vehicle. The synthesis of **10** was initiated by treatment of alcohol **6** with thiophenol to generate dithiomaleimide **8**, which was then reacted with 4-nitrophenyl chloroformate to give activated carbonate **9**. Simple treatment of **9** with DOX·HCl under basic conditions yielded desired linker-drug construct **10** in excellent overall yield (Scheme 3).

**Scheme 3:**
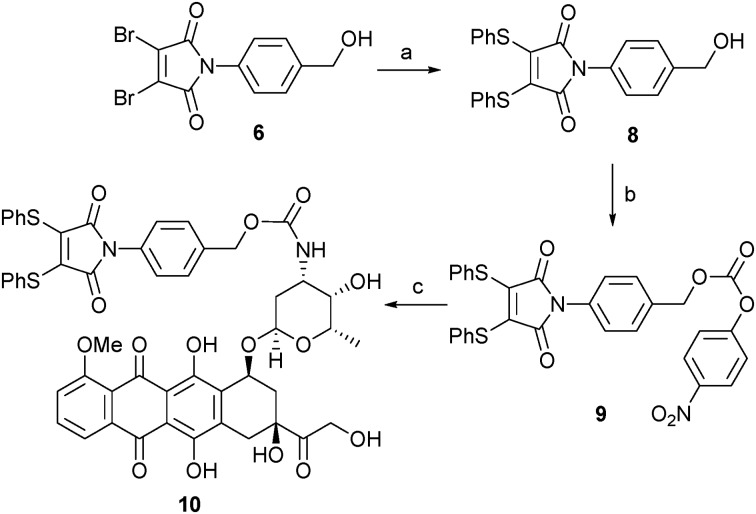
Synthesis of thiomaleamate–PABC–DOX construct **10**: (a) PhSH, NEt_3_, CH_2_Cl_2_, 98%; (b) PNPC, py, CH_2_Cl_2_, 72%; (c) DOX·HCl, NEt_3_, NMP, 99%.

Trastuzumab (Herceptin™) is a monoclonal IgG1 antibody that targets the HER2/neu receptor, is known to be able to internalise and has been used successfully in the treatment of HER2-positive breast cancer.^13^ It is also the antibody component of trastuzumab emtansine (Kadcycla™), a recently FDA-approved ADC therapy for the same indication.^14^ We thus rationalised that the Fab fragment of trastuzumab, **11**, would be an appealing antibody fragment system on which to evaluate novel drug-linker **10**. The Fab fragment contains the antigen-binding region of the full antibody and consists of one constant and one variable region on each of the light and heavy polypeptide chains. The light and heavy chains are connected *via* a single interchain disulfide bond (Scheme 4). It was envisaged that functional bridging of this interchain disulfide would yield a homogeneous ADC product.

**Scheme 4:**
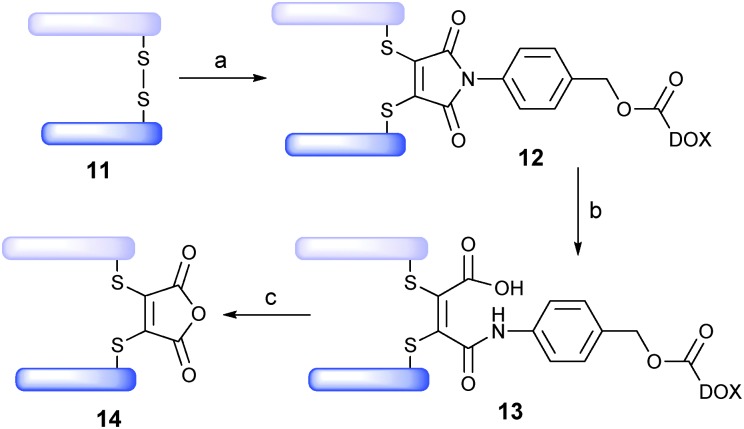
Assembly/cleavage study of Fab ADC **13**: (a) TCEP, pH 8.0, 37 °C, 1.5 h, then **10**, 37 °C, 1 h; (b) pH 7.4, 20 h; (c) pH 4.5, 72 h.

Typically, a Fab fragment can be obtained from a full IgG1 antibody following a papain digest.^15^ However, we discovered that when this approach was applied to trastuzumab it yielded two products, indicating that papain cleaves trastuzumab at two sites (see ESI†). To combat this, we developed an elegant alternative protocol to generate Fab fragment **11**, which involved sequential digests with pepsin (yielding the F(ab′)_2_ fragment) and papain to afford the desired Fab fragment as a single product, following ultrafiltration, in 64% overall yield.

With trastuzumab-Fab **11** in-hand, we treated it with tris(2-carboxyethyl)-phosphine (TCEP, 3 eq.) to affect reduction of the interchain disulfide. Subsequent treatment with drug-linker **10** (5 eq., pH 8, 37 °C, 1 h) yielded bioconjugate **12** in near quantitative yield (LCMS expected 48 414, observed 48 413). The pH was then adjusted to physiological pH (pH 7.4) and bioconjugate **12** converted to ADC **13** (37 °C, 20 h) (LCMS expected 48 432, observed 48 433). To our delight, prolonged incubation of ADC **13** (pH 7.4, 37 °C, 3 days) demonstrated stability of the linker under physiological conditions. Moreover, incubation of ADC **13** at lysosomal pH and temperature (pH 4.5, 37 °C, 72 h) showed progressive cleavage of the cargo, yielding **14**.

We then sought to appraise the impact on antibody–antigen binding of conjugating DOX to trastuzumab-Fab **11** using our new linker. We rationalised that, as the interchain disulfide is distal from the antigen-binding site of the Fab fragment, that the impact should be minimal. To assess this, the binding profile of ADC **13** was evaluated against unmodified Fab fragment **11** and a Fab fragment that had been subjected to the reaction conditions employed in assembling **13** minus the reducing agent and construct **10** (*i.e.* processed Fab) using ELISA. Encouragingly, comparable antigen binding was observed across all three constructs (Fig. 1).

**Fig. 1:**
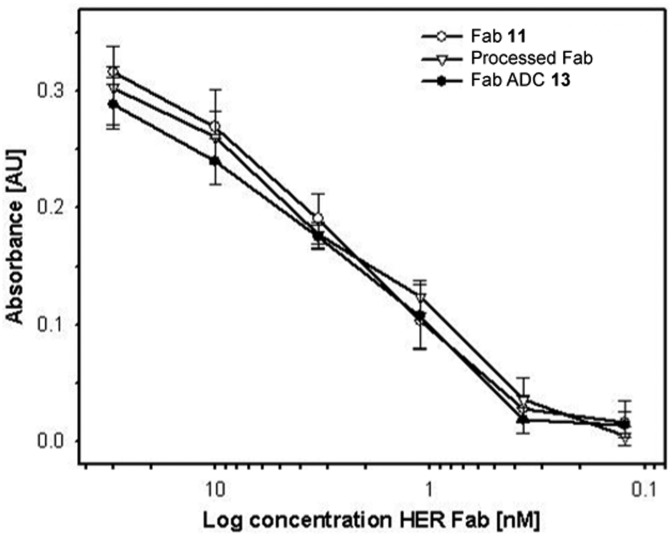
ELISA analysis of Fab **11**, processed Fab and Fab ADC **13** binding to the HER2 antigen.

The ADC concept allows potent small-molecule therapeutics to be targeted to a site of disease with minimal off-target toxicity. This concept is now validated following FDA approval of Kadcycla™ and Adcetris™, for the treatment of HER2-positive breast cancer and Hodgkin Lymphoma respectively.^14,16^ However, there is a recognised requirement for conjugation chemistries that yield more homogeneous ADC products and that facilitate release of the payload once the ADC has been internalised into its target cell. We have demonstrated a new approach to antibody-conjugation that allows site-selective drug attachment, *via* functional re-bridging of the native interchain disulfide bond of a Fab fragment, and release of the drug payload under mildly acidic, lysosomal pH. We have also demonstrated that antigen binding of the antibody is retained following drug conjugation. We will now seek to evaluate this linker methodology through assembly and evaluation of a range of novel ADCs targeted towards cancer treatment.

The authors gratefully acknowledge the EPSRC, BBSRC, Wellcome Trust and UCLB for support of our programme.
