# Supplementary material for: Acid-cleavable thiomaleamic acid linker for homogeneous antibody–drug conjugation
Source: Chem Commun (Camb). 2013 Aug 9;49(74):8187–9. doi: 10.1039/c3cc45220d (PMC3763782; doi:10.1039/c3cc45220d)
Supplement: Supplementary file 1 [file CC-049-c3cc45220d-s001.pdf]

This journal is © The Royal Society of Chemistry 2013

Acid-cleavable thiomaleamic acid linker for homogeneous antibody-drug conjugation

Lourdes Castañeda,[a] Antoine Maruani,[a] Felix F. Schumacher,[a] Enrique Miranda,[b] Vijay Chudasama,[a] Kerry A. Chester,[b] James R. Baker,[a] Mark E. B. Smith,*[a] Stephen Caddick*[a]

[a] *Department of Chemistry, University College London, 20 Gordon Street, London, WC1H OAJ, UK;* [b] *UCL Cancer Institute, 72 Huntley Street, London, WC1E 6BT, UK.*

Tel: +44 (0)20 7679 3721; E-mail: **m.e.b.smith@ucl.ac.uk**

Tel: +44 (0)20 3108 5071; Fax: +44 (0)20 7679 7463; E-mail: **vpenterprise@ucl.ac.uk**

General Experimental

All reagents were purchased from Sigma-Aldrich or AlfaAesar and were used as received without further purification. Where described below petrol refers to petrol (b.p. 40-60 °C). All reactions were monitored by thin-layer chromatography (TLC) on pre-coated SIL G/UV254 silica gel plates (254 μm) purchased from VWR. Flash column chromatography was carried out with Kiesegel 60M 0.04/0.063 mm (200-400 mesh) silica gel. ^{1}H and ^{13}C NMR spectra were recorded at ambient temperature on a Bruker Avance 600 instrument operating at a frequency of 600 MHz for ^{1}H and 150 MHz for ^{13}C in a deuterated solvent as described below. The chemical shifts (δ) for ^{1}H and ^{13}C are quoted relative to residual signals of the solvent on the ppm scale. ^{1}H NMR peaks are reported as singlet (s), doublet (d), triplet (t), quartet (q), quintet (quintet), broad (br) or multiplet (m). Coupling constants (J values) are reported in Hertz (Hz) and are H-H coupling constants unless otherwise stated. Signal multiplicities in ^{13}C NMR were determined using the distortionless enhancement by phase transfer (DEPT) spectral editing technique. Infrared spectra were obtained on a Perkin Elmer Spectrum 100 FTIR Spectrometer operating in ATR mode with frequencies given in reciprocal centimetres (cm^{-1}). Melting points were measured with a Gallenkamp apparatus and are uncorrected. Mass spectra were obtained on a VG70-SE mass spectrometer. Optical rotations were measured using a Perkin Elmer 343 polarimeter.

LC-MS was performed on protein samples using a Thermo Scientific uPLC connected to MSQ Plus Single Quad Detector (SQD). Column: Hypersil Gold C4, 1.9 μm 2.1 x 50 mm. Wavelength: 254 nm. Mobile Phase: 99:1 Water:MeCN (0.1% formic acid) to 1:9 Water: MeCN (0.1% formic acid) gradient over 4 min. Flow Rate: 0.3 mL/min. MS Mode: ES^{+}. Scan Range: m/z = 500-2000. Scan time: 1.5 s. Data obtained in continuum mode. The electrospray source of the MS was operated with a capillary voltage of 3.5 kV and a cone voltage of 50 V. Nitrogen was used as the nebulizer and desolvation gas at a total flow of 600 L/h. Ion series were generated by integration of the total ion chromatogram (TIC) over the 3.5-5.0 min range. Total mass spectra for protein samples were reconstructed from the ion series using the pre-installed ProMass software.

This journal is © The Royal Society of Chemistry 2013

3,4-Dibromo-furan-2,5-dione[1]

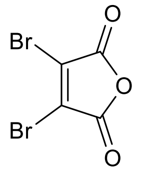

A mixture of maleic anhydride (1.50 g, 15.3 mmol), $AlCl_3$ (28 mg, 0.21 mmol) and Br_2 (1.57 mL, 4.89 g, 30.6 mmol) was heated at 160 ºC in a sealed ampoule for 20 h. After this time, the reaction mixture was allowed to cool to room temperature, and diluted with EtOAc (25 mL). The reaction mixture was then filtered and the solid washed thoroughly with EtOAc (3 × 25 mL). The filtrate was then concentrated *in vacuo* to afford 3,4-dibromo-furan-2,5-dione as a yellow solid (3.25 g, 12.8 mmol, 83%): m.p. 107-110 °C (*lit. m.p. 113-114 °C*)[1]; ^{13}C NMR (MeOD, 150 MHz) δ 164.4 (C), 125.9 (C); IR (solid) 1769, 1706, 1590 cm^{-1}.

3,4-Dibromo-1-phenyl-pyrrole-2,5-dione 1[1]

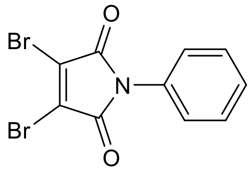

To a solution of 3,4-dibromo-furan-2,5-dione (0.50 g, 1.97 mmol) in AcOH (25 mL) was added aniline (0.18 mL, 1.97 mmol), and the reaction mixture stirred at room temperature for 3 h and then at 130 °C for 90 min. After this time, the solvents were removed *in vacuo*, with the residual traces of AcOH removed by azeotropic distillation using toluene (3 × 25 mL). The crude residue was purified by flash column chromatography (5% EtOAc/petrol) to afford 3,4-dibromo-1-phenyl-pyrrole-2,5-dione **1** as a pale yellow solid (0.37 g, 1.12 mmol, 57%): m.p. 158-160 °C (*lit. m.p. 162-163 °C*)[1]; ^{1}H NMR ($CDCl_3$, 600 MHz) δ 7.48-7.47 (2H, m, Ar*H*), 7.44-7.40 (1H, m, Ar*H*), 7.33 (2H, d, *J* = 7.2 Hz, Ar*H*); ^{13}C NMR ($CDCl_3$, 150 MHz) δ 163.0 (C), 132.8 (C), 130.8 (C), 129.5 (CH), 128.8 (CH), 126.2 (CH); IR (solid) 3058, 1727, 1715, 1646, 1610, 1598, 1501 cm^{-1}; LRMS (EI) 333 (50, [M^{81}Br^{81}Br]$^+$), 331 (100, [M^{81}Br ^{79}Br]$^+$), 329 (50, [M^{79}Br ^{79}Br]$^+$); HRMS (EI) calcd for $C_{10}H_5O_2NBr_2$ [M^{79}Br ^{79}Br]$^+$ 328.8682, observed 328.8685.

3,4-Bis-ethylsulfanyl-1-phenyl-pyrrole-2,5-dione 2

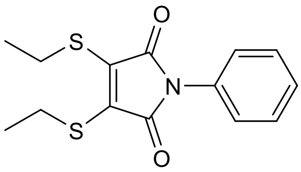

To a solution of 3,4-dibromo-1-phenyl-pyrrole-2,5-dione **1** (237 mg, 0.72 mmol) in CH_2Cl_2 (30 mL) was added ethanethiol (0.11 mL, 1.50 mmol) and NEt_3 (0.21 mL, 1.50 mmol), and the reaction mixture stirred at room temperature for 15 min. After this time, the solvents were removed *in vacuo* and the crude residue purified by flash column chromatography (5% EtOAc/petrol) to afford 3,4-bis-ethylsulfanyl-1-phenyl-pyrrole-2,5-dione **2** as a yellow oil (195 mg, 0.66 mmol, 93%): ^{1}H NMR ($CDCl_3$, 600 MHz) δ 7.48-7.44 (2H, m, Ar*H*), 7.37-7.33 (3H, m, Ar*H*), 3.37 (4H, q, *J* = 7.4 Hz, CH_3CH_2S), 1.38 (6H, t, *J* = 7.4 Hz, CH_3CH_2S); ^{13}C NMR ($CDCl_3$, 150 MHz) δ 165.5 (C), 135.7 (C), 131.6 (C), 129.2 (CH), 128.0 (CH), 126.2 (CH), 26.5 (CH_2), 15.8 (CH_3); IR (neat) 2965, 2926, 2851, 1706, 1598, 1501 cm^{-1}; LRMS (EI) 293 (100, [M]$^+$); HRMS (EI) calcd for $C_{14}H_{15}O_2NS_2$ [M]$^+$ 293.0539, observed 293.0540.

Electronic Supplementary Material (ESI) for Chemical Communications
This journal is © The Royal Society of Chemistry 2013

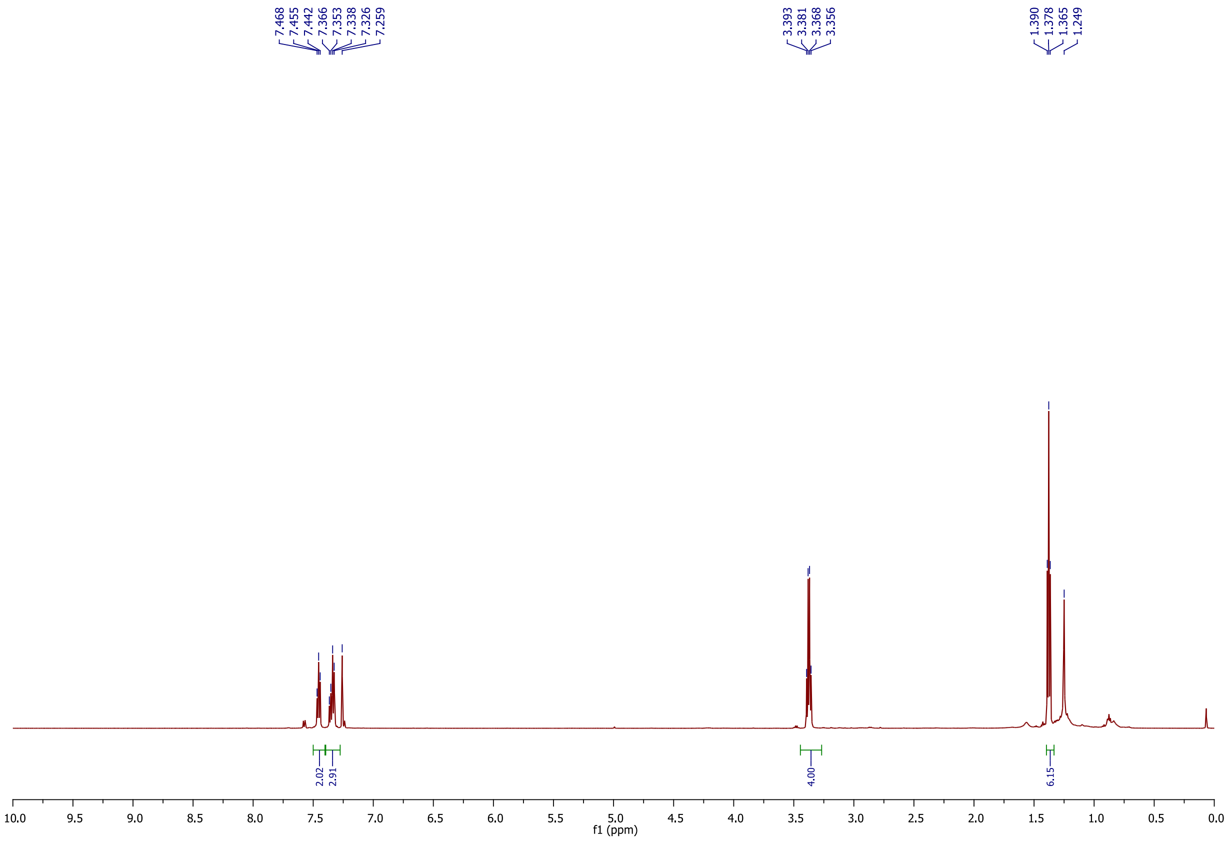

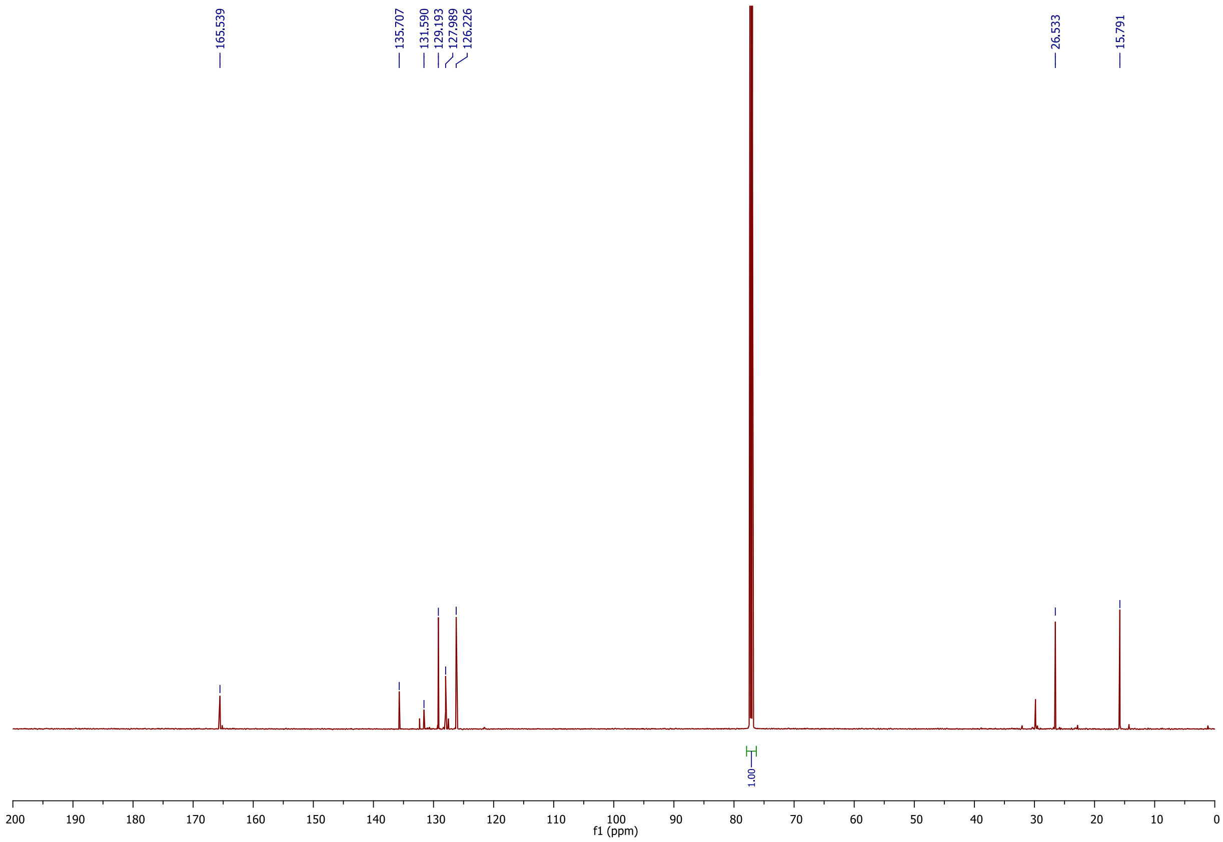

This journal is © The Royal Society of Chemistry 2013

Lithium 2,3-bis-ethylsulfanyl-3-phenylcarbamoyl-acrylate 3

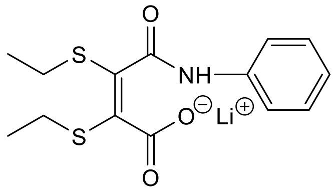

To a solution of 3,4-bis-ethylsulfanyl-1-phenyl-pyrrole-2,5-dione **2** (19.0 mg, 0.065 mmol) in a 1:1 mixture of CD_3OD:D_2O (1 mL:1 mL) was added LiOH.H_2O (146 mg, 3.48 mmol) and the reaction mixture stirred at room temperature for 24 h to afford lithium 2,3-bis-ethylsulfanyl-3-phenylcarbamoyl-acrylate **3**: ^{1}H NMR (CD_3OD/D_2O, 600 MHz) δ 7.46 (2H, d, J = 7.8 Hz, ArH), 7.38 (2H, t. J = 7.8 Hz, ArH), 7.18 (1H, t, J = 7.8 Hz, ArH), 2.90 (2H, q, J = 7.5 Hz, CH_3CH_2S), 2.80 (2H, q, J = 7.5 Hz, CH_3CH_2S), 1.31 (3H, t, J = 7.5 Hz, CH_3CH_2S), 1.29 (3H, t, J = 7.5 Hz, CH_3CH_2S); ^{13}C NMR (CD_3OD/D_2O, 150 MHz) δ 171.7 (C), 166.0 (C), 156.9 (C), 139.6 (C), 130.3 (CH), 126.3 (C), 122.7 (CH), 119.9 (CH), 28.9 (CH_2), 28.0 (CH_2), 15.7 (CH_3), 15.3 (CH_3).

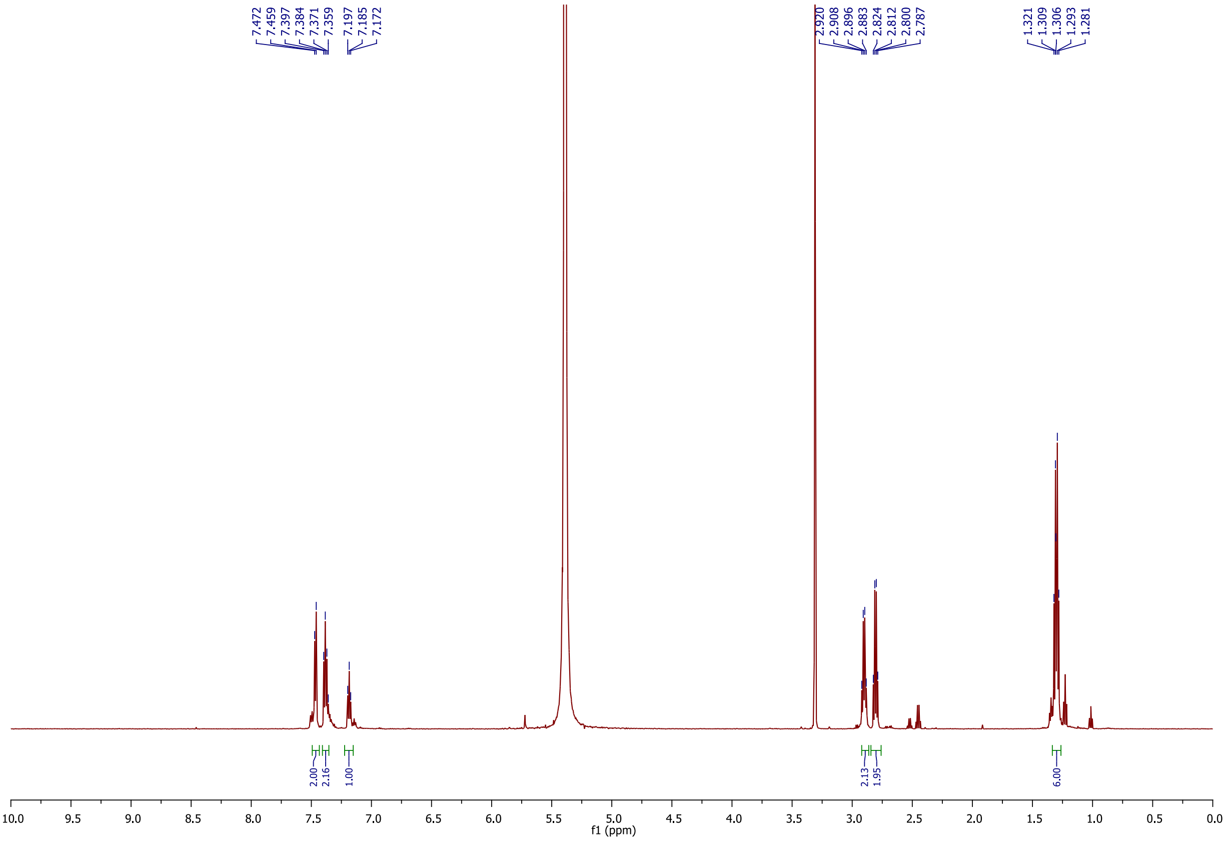

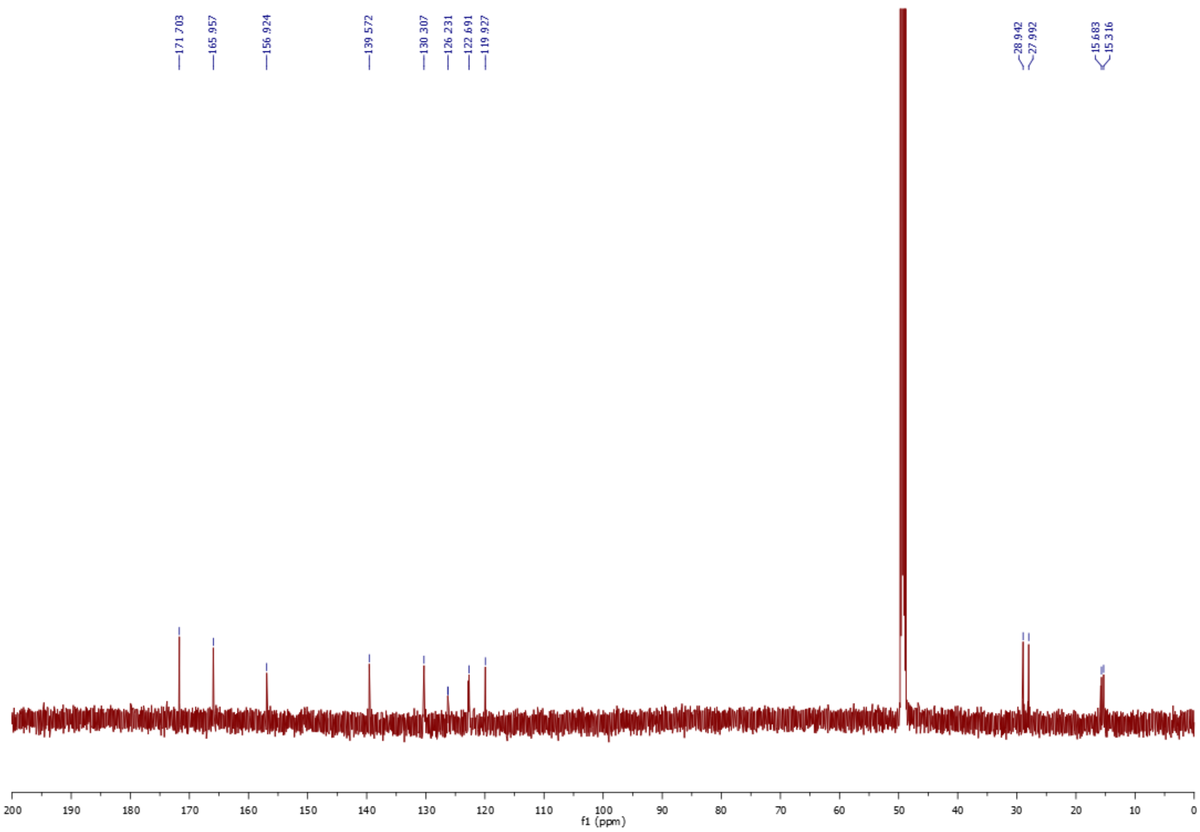
171.703
165.957
156.924
139.572
130.307
126.231
122.691
119.927
28.942
27.992
15.683
15.316
200 190 180 170 160 150 140 130 120 110 100 90 80 70 60 50 40 30 20 10 0
f1 (ppm)

This journal is © The Royal Society of Chemistry 2013

3,4-Bis-ethylsulfanyl-furan-2,5-dione 4

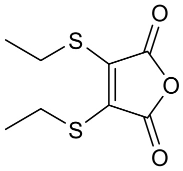

To a solution of lithium 2,3-bis-ethylsulfanyl-3-phenylcarbamoyl-acrylate **3** (0.065 mmol) in a 1:1 mixture of CD_3OD:D_2O (1 mL:1 mL) was added 2M HCl and the reaction mixture acidified to pH 4. After this, 3,4-bis-ethylsulfanyl-furan-2,5-dione **4** was extracted from the reaction mixture into EtOAc (3 × 20 mL), washed with sat. NaCl, dried ($MgSO_4$), filtered and the solvents removed *in vacuo* to afford 3,4-bis-ethylsulfanyl-furan-2,5-dione **4** as an oil (14.0 mg, 0.065 mmol, >99%% over two steps): ^1H NMR ($CDCl_3$, 600 MHz) δ 3.37 (4H, q, J = 7.4 Hz, CH_3CH_2S), 1.36 (6H, t, J = 7.4 Hz, CH_3CH_2S); ^{13}C NMR ($CDCl_3$, 150 MHz) δ 160.4 (C), 136.9 (C), 26.4 (CH_2), 15.7 (CH_3); IR (neat) 2969, 2930, 1761, 1518 cm^{-1}; LRMS (EI) 218 (100, $[M]^+$); HRMS (EI) calcd for $C_8H_{10}O_3S_2$ $[M]^+$ 218.0066, observed 218.0071.

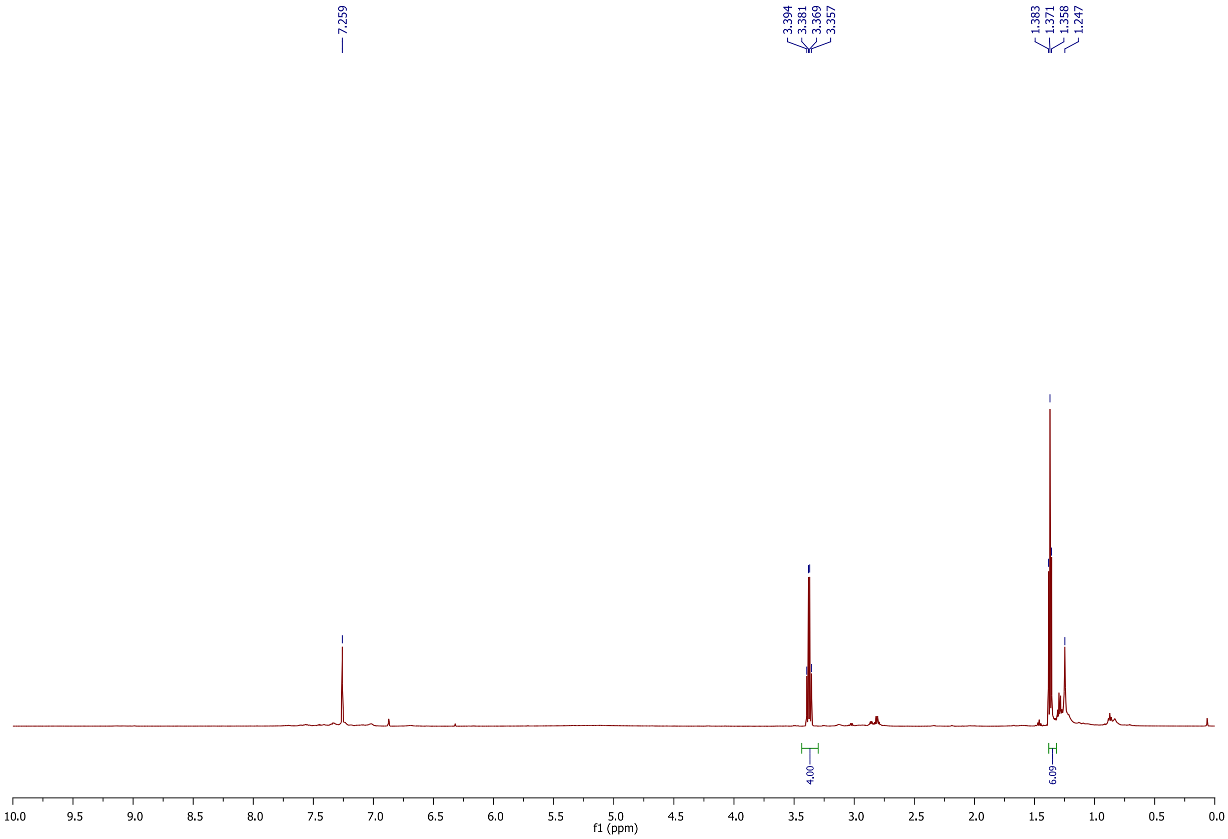

This journal is © The Royal Society of Chemistry 2013

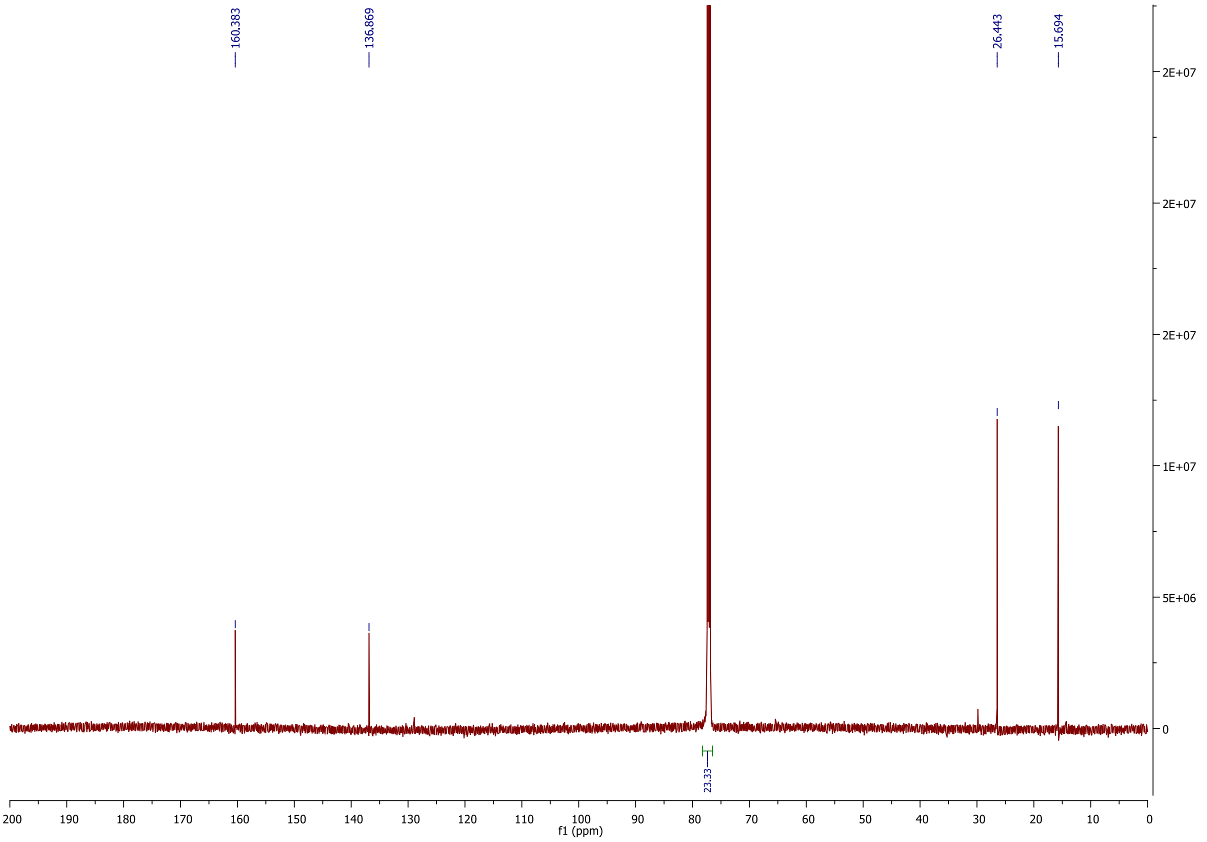

3,4-Dibromo-2,5-dioxo-2,5-dihydro-pyrrole-1-carboxylic acid methyl ester 5[2]

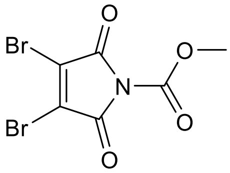

To a solution of 3,4-dibromo-furan-2,5-dione (1.0 g, 3.9 mmol) and *N*-methylmorpholine (0.43 mL, 3.9 mmol) in THF (35 mL) was added methyl chloroformate (0.30 mL, 3.9 mmol) and the reaction mixture stirred at room temperature for 20 min. After this time, CH_2Cl_2 (40 mL) was added, the organic phase washed with H_2O (3 × 40 mL), dried ($MgSO_4$) and the solvent removed *in vacuo* to afford 3,4-dibromo-2,5-dioxo-2,5-dihydro-pyrrole-1-carboxylic acid methyl ester **5** as a pink powder (1.18 g, 3.8 mmol, 97%): m.p. 114-116 °C (*lit. m.p. 115-118 °C*)[2]; ^{1}H NMR ($CDCl_3$, 600 MHz) δ 4.00 (3H, s, CH_3); ^{13}C NMR ($CDCl_3$, 150 MHz) δ 159.3 (C), 147.0 (C), 131.5 (C), 54.9 (CH_3); IR (solid) 3236, 2962, 1809, 1769, 1730, 1602 cm^{-1}; LRMS (CI) 314 (50, $[M^{81}Br^{81}Br + H]^+$), 312 (100, $[M^{81}Br\,^{79}Br]^+$), 310 (50, $[M^{79}Br\,^{79}Br]^+$); HRMS (EI) calcd for $C_6H_3O_4NBr_2$ $[M^{79}Br^{79}Br]^+$ 310.8423, observed: 310.8427.

3,4-Dibromo-1-(4-hydroxymethyl-phenyl)-pyrrole-2,5-dione 6[2]

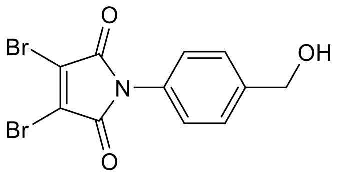

To a solution of 3,4-dibromo-2,5-dioxo-2,5-dihydro-pyrrole-1-carboxylic acid methyl ester **5** (300 mg, 0.965 mmol) in CH_2Cl_2 (42 mL) was added 4-aminobenzyl alcohol (119 mg, 0.965

This journal is © The Royal Society of Chemistry 2013

mmol), and the reaction mixture stirred at room temperature for 2 h. After this time, the solvents were removed *in vacuo* and the crude residue purified by flash column chromatography (50% EtOAc/petrol) to afford 3,4-dibromo-1-(4-hydroxymethyl-phenyl)-pyrrole-2,5-dione **6** as a yellow solid (345 mg, 0.955 mmol, 99%): m.p. 215-217 °C (*lit. m.p. 217-220 °C*)[2]; ^{1}H NMR ($CDCl_3$, 600 MHz) δ 7.49 (2H, d, *J* = 8.4 Hz, Ar*H*), 7.33 (2H, d, *J* = 8.4 Hz, Ar*H*), 4.75 (2H, s, C*H*$_2$OH); ^{13}C NMR ($CDCl_3$, 150 MHz) δ 163.0 (C), 141.6 (C), 130.2 (C), 130.0 (C), 127.8 (CH), 126.3 (CH), 64.7 (CH_2); IR (solid) 3364, 1732, 1714, 1610, 1519 cm^{-1}; LRMS (EI) 361 (50, [M^{81}Br^{81}Br]$^+$), 359 (100, [M^{81}Br ^{79}Br]$^+$), 357 (50, [M^{79}Br ^{79}Br]$^+$); HRMS (EI) calcd for $C_{11}H_7O_3NBr_2$ [M^{79}Br^{79}Br]$^+$ 358.8787, observed: 358.8798.

3,4-Bis-ethylsulfanyl-1-(4-hydroxymethyl-phenyl)-pyrrole-2,5-dione

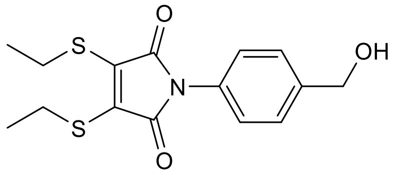

To a solution of 3,4-dibromo-1-(4-hydroxymethyl-phenyl)-pyrrole-2,5-dione **6** (315 mg, 0.872 mmol) in CH_2Cl_2 (40 mL) was added ethanethiol (0.18 mL, 2.33 mmol) and NEt_3 (0.33 mL, 2.33 mmol) and the reaction mixture stirred at room temperature for 1 h. After this time, the reaction mixture was concentrated *in vacuo* and the crude residue purified by flash column chromatography (50% EtOAc/petrol) to afford 3,4-bis-ethylsulfanyl-1-(4-hydroxymethyl-phenyl)-pyrrole-2,5-dione as a yellow solid (242 mg, 0.748 mmol, 86%): m.p. 50-52 ºC; ^{1}H NMR ($CDCl_3$, 600 MHz) δ 7.45 (2H, d, *J* = 8.4 Hz, Ar*H*), 7.33 (2H, d, *J* = 8.4 Hz, Ar*H*), 4.72 (2H, s, C*H*$_2$OH), 3.37 (4H, q, *J* = 7.4 Hz, CH_3C*H*$_2$S), 1.37 (6H, t, *J* = 7.4 Hz, C*H*$_3$$CH_2$S); ^{13}C NMR ($CDCl_3$, 150 MHz) δ 165.5 (C), 140.7 (C), 135.7 (C), 130.9 (C), 127.6 (CH), 126.3 (CH), 64.9 (CH_2), 26.5 (CH_2), 15.8 (CH_3); IR (solid) 3381, 2967, 2928, 2869, 1702, 1514 cm^{-1}; LRMS (EI) 323 (100, [M]$^+$); HRMS (EI) calcd for $C_{15}H_{17}O_3NS_2$ [M]$^+$ 323.0644, observed: 323.0634.

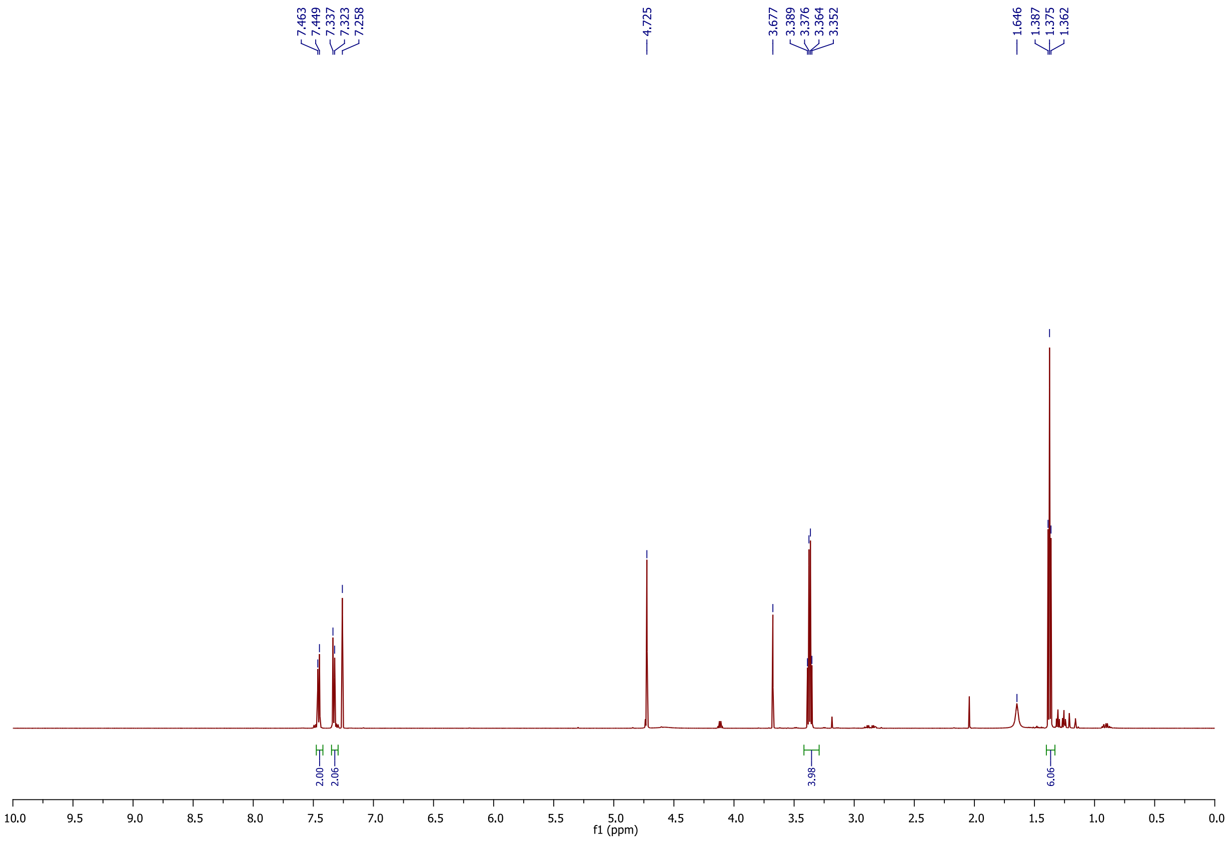
7.463
7.449
7.337
7.323
7.258
4.725
3.677
3.389
3.376
3.364
3.352
1.646
1.387
1.375
1.362
2.00
2.06
3.98
6.06
10.0 9.5 9.0 8.5 8.0 7.5 7.0 6.5 6.0 5.5 5.0 4.5 4.0 3.5 3.0 2.5 2.0 1.5 1.0 0.5 0.0
f1 (ppm)

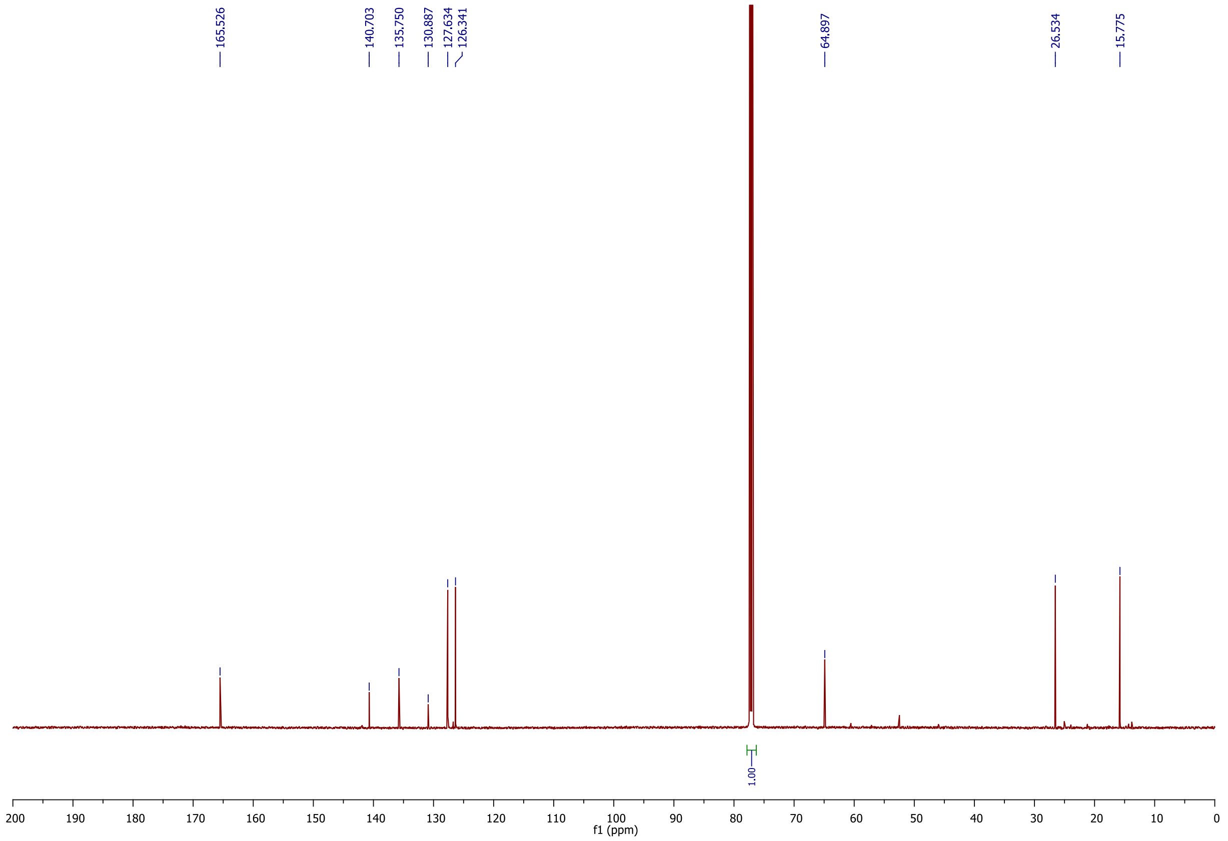
165.526
140.703
135.750
130.887
127.634
126.341
64.897
26.534
15.775
1.00
200 190 180 170 160 150 140 130 120 110 100 90 80 70 60 50 40 30 20 10 0
f1 (ppm)

Phenyl-carbamic acid 4-(3,4-bis-ethylsulfanyl-2,5-dioxo-2,5-dihydro-pyrrol-1-yl)-benzyl ester 7

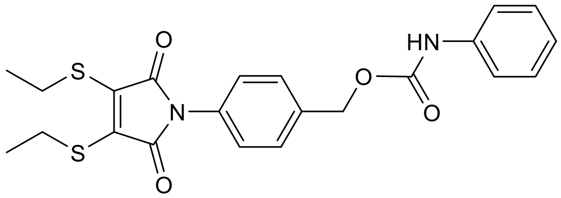

To a solution of 3,4-bis-ethylsulfanyl-1-(4-hydroxymethyl-phenyl)-pyrrole-2,5-dione (100 mg, 0.309 mmol) in CH_2Cl_2 (14 mL) was added phenylisocyanate (0.035 mL, 0.322 mmol) and NEt_3 (0.091 mL, 0.653 mmol), and the reaction mixture stirred at room temperature for 2 days. After this time, the reaction mixture was concentrated *in vacuo* and the crude residue purified by flash column chromatography (20% EtOAc/petrol) to afford phenyl-carbamic acid 4-(3,4-bis-ethylsulfanyl-2,5-dioxo-2,5-dihydro-pyrrol-1-yl)-benzyl ester **7** as a yellow solid (84 mg, 0.190 mmol, 62%): m.p. 97-99 ºC; ^{1}H NMR ($CDCl_3$, 600 MHz) δ 7.49 (2H, d, *J* = 8.3 Hz, Ar*H*CH_2O), 7.39-7.35 (4H, m, Ar*H*CH_2O and Ar*H*NH), 7.32-7.30 (2H, m, Ar*H*NH), 7.07 (1H, t, *J* = 7.4 Hz, Ar*H*NH), 6.66 (1H, br s, N*H*), 5.22 (2H, s, C*H*$_2$OCONH), 3.37 (4H, q, *J* = 7.4 Hz, CH_3C*H*$_2$S), 1.37 (6H, t, *J* = 7.4 Hz, C*H*$_3$$CH_2$S); ^{13}C NMR ($CDCl_3$, 150 MHz) δ 165.4 (C), 153.2 (C), 137.7 (C), 135.8 (C), 131.6 (C), 129.2 (CH), 129.1 (CH), 126.3 (CH), 126.2 (CH), 123.7 (CH), 118.8 (C), 66.4 (CH_2), 26.5 (CH_2), 15.8 (CH_3); IR (solid) 3374, 2964, 2928, 1733, 1705, 1598, 1532 cm^{-1}; LRMS (ESI) 465 (100, $[M+Na]^+$); HRMS (ESI) calcd for $C_{22}H_{22}N_2O_4NaS_2$ $[M+Na]^+$ 465.0919, observed: 465.0923.

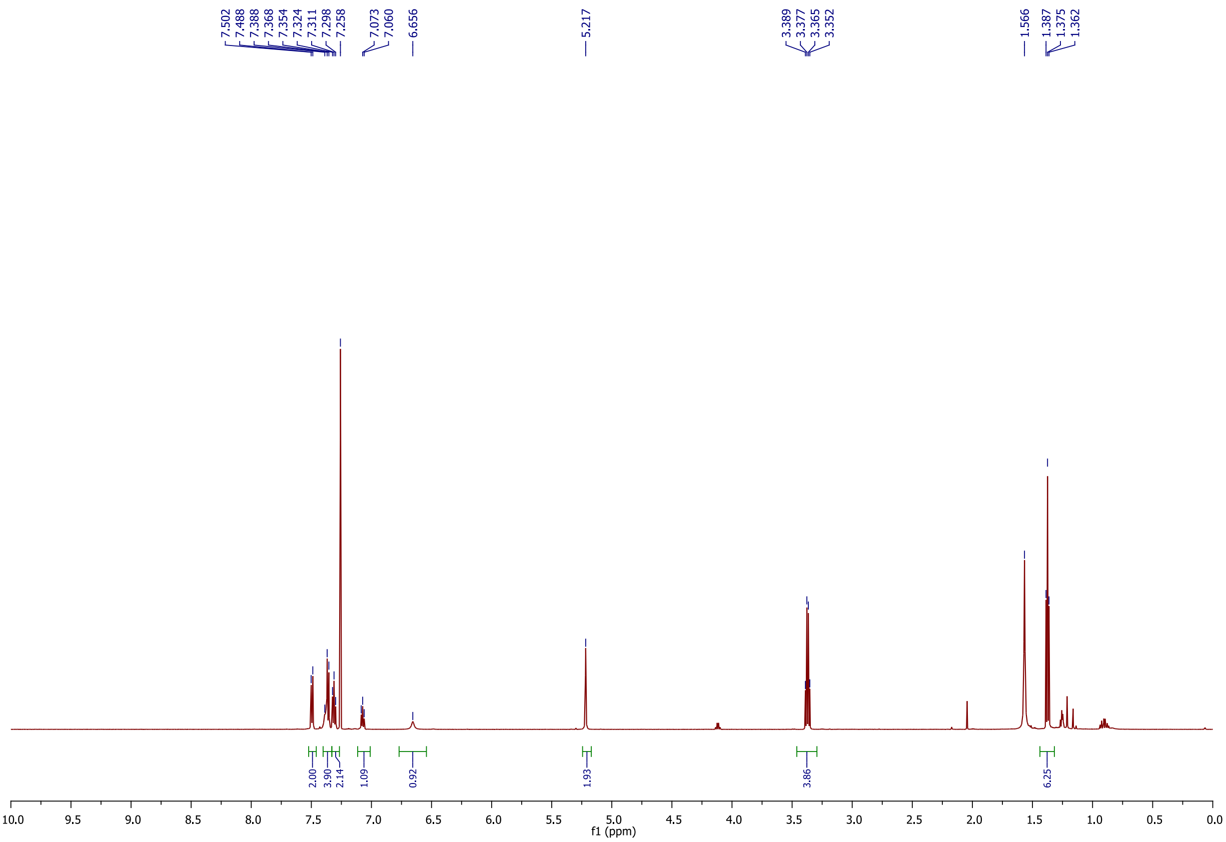

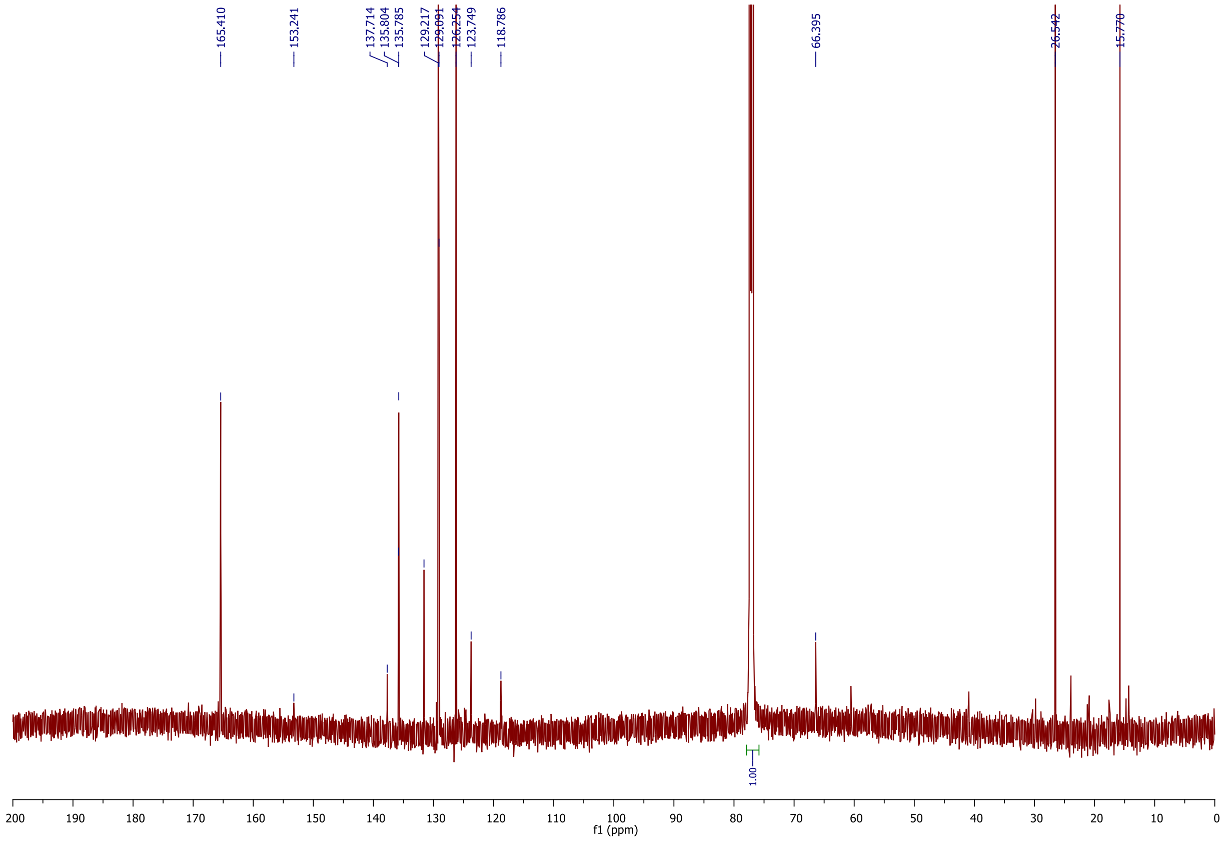
165.410
153.241
137.714
135.804
135.785
129.217
129.091
126.254
123.749
118.786
66.395
26.542
15.770
1.00
200
190
180
170
160
150
140
130
120
110
100
90
80
70
60
50
40
30
20
10
0
f1 (ppm)

3,4-Bis-ethylsulfanyl-furan-2,5-dione 4 and aniline

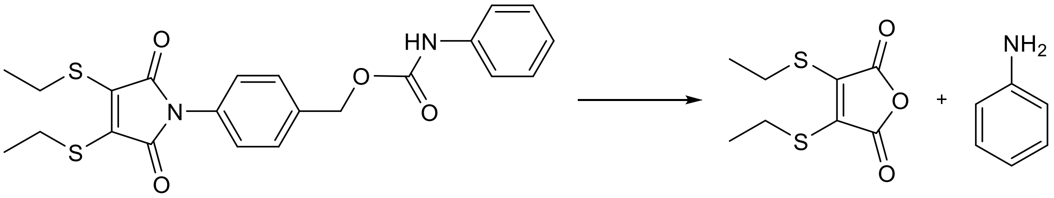

To a solution of phenyl-carbamic acid 4-(3,4-bis-ethylsulfanyl-2,5-dioxo-2,5-dihydro-pyrrol-1-yl)-benzyl ester **7** (19.5 mg, 0.044 mmol) in a 1:1 mixture of CD_3OD:D_2O (0.8 mL:0.8 mL) was added LiOH.H_2O (102 mg, 2.44 mmol) and the reaction mixture stirred at room temperature for 2 h. After this time, 2M HCl was added and the reaction mixture acidified to pH 4. Then, 3,4-bis-ethylsulfanyl-furan-2,5-dione **4** was extracted from the reaction mixture into EtOAc (3 × 20 mL), washed with sat. NaCl, dried ($MgSO_4$), filtered and the solvents removed *in vacuo* to afford 3,4-bis-ethylsulfanyl-furan-2,5-dione **4** as an oil (9.6 mg, 0.044 mmol, >99%). Data matched that described above.

The pH 4, aqueous solution was neutralised by addition of 1M NaOH. Then, the organic materials were extracted into $CHCl_3$ (3 × 5 mL), dried ($MgSO_4$), filtered and the solvent was removed *in vacuo*. The crude residue was purified by flash column chromatography (10% EtOAc/petrol) to afford aniline as an oil (3.9 mg, 0.042 mmol, 95%). ^{1}H NMR ($CDCl_3$, 600 MHz) δ 7.16 (2H, t, *J* = 8.0 Hz, Ar*H*), 6.77 (1H, t, *J* = 8.0 Hz, Ar*H*), 6.70 (2H, d, *J* = 8.0 Hz, Ar*H*), 3.84 (2H, br s, NH_2); ^{13}C NMR ($CDCl_3$, 150 MHz) δ 146.2 (C), 129.4 (CH), 118.9 (CH), 115.4 (CH); IR (neat) 3445, 3354, 3210, 3068, 3032, 1619, 1602, 1497 cm^{-1}.

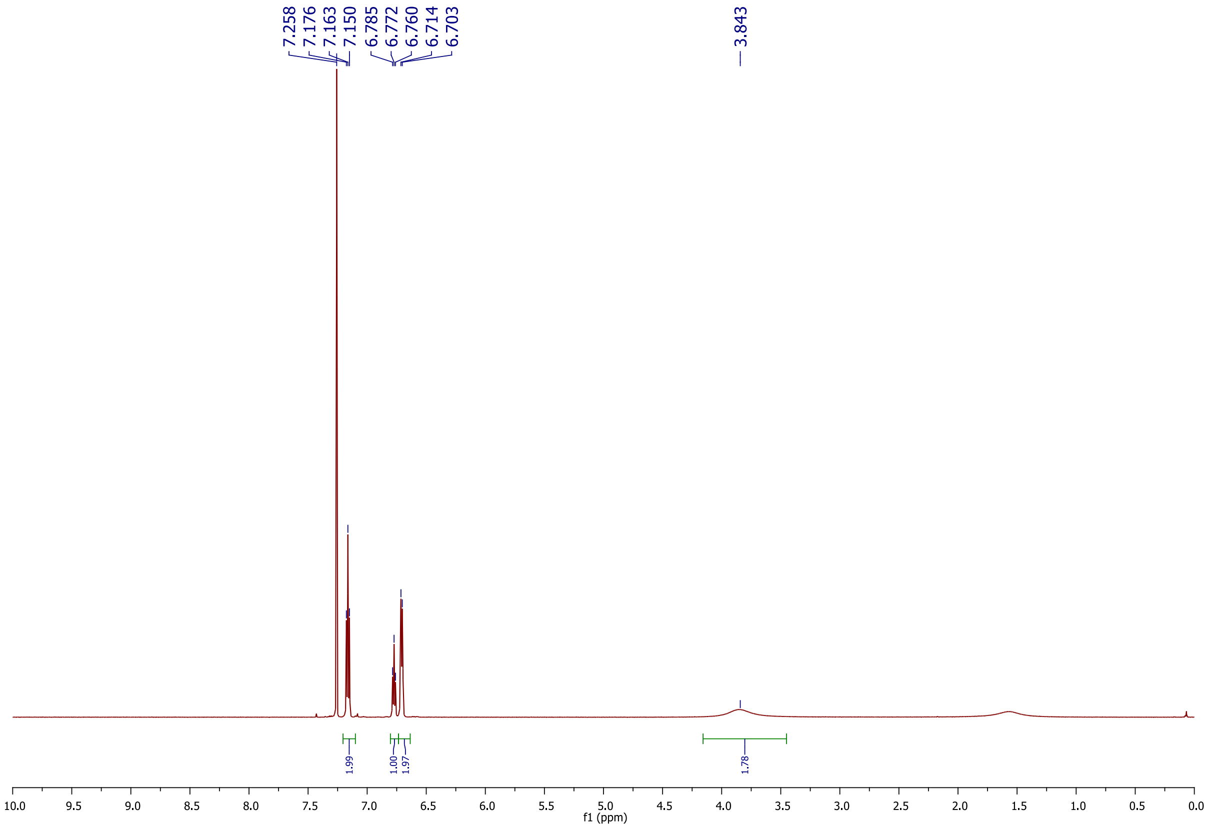

This journal is © The Royal Society of Chemistry 2013

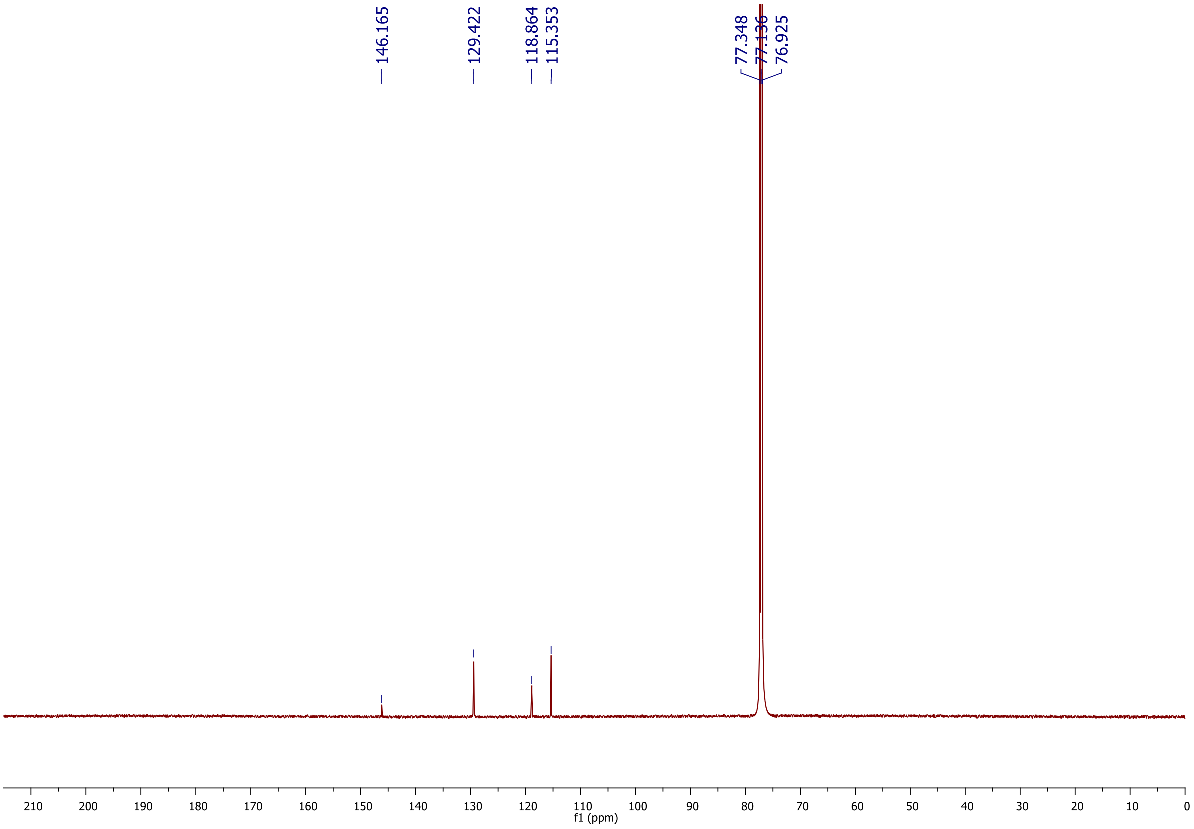

1-(4-Hydroxymethyl-phenyl)-3,4-bis-phenylsulfanyl-pyrrole-2,5-dione 8

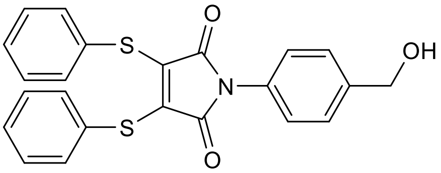

To a solution of 3,4-dibromo-1-(4-hydroxymethyl-phenyl)-pyrrole-2,5-dione **6** (139 mg, 0.385 mmol) in CH_2Cl_2 (18 mL) was added NEt_3 (0.145 mL, 1.04 mmol) and phenylthiol (0.08 mL, 0.809 mmol), and the reaction mixture stirred at room temperature for 30 min. After this time, the reaction mixture was concentrated *in vacuo* and the crude residue purified by flash column chromatography (20% EtOAc/petrol) to afford 1-(4-hydroxymethyl-phenyl)-3,4-bis-phenylsulfanyl-pyrrole-2,5-dione **8** as a yellow solid (159 mg, 0.378 mmol, 98%): m.p. 111-113 ºC; ^{1}H NMR ($CDCl_3$, 600 MHz) δ 7.46 (2H, d, J = 8.4 Hz, Ar*H*CH_2OH), 7.32 (2H, d, J = 8.4 Hz, Ar*H*CH_2OH), 7.31-7.26 (10H, m, Ar*H*S), 4.70 (2H, s, C*H*$_2$OH); ^{13}C NMR ($CDCl_3$, 150 MHz) δ 165.8 (C), 140.7 (C), 135.8 (C), 132.2 (CH), 130.8 (C), 129.2 (CH), 128.8 (C), 128.7 (CH), 127.6 (CH), 126.1 (CH), 64.9 (CH_2); IR (solid) 3388, 3057, 2928, 2874, 1705, 1515 cm^{-1}; LRMS (EI) 419 (100, $[M]^+$); HRMS (EI) calcd for $C_{23}H_{17}O_3NS_2$ $[M]^+$ 419.0650, observed: 419.0653.

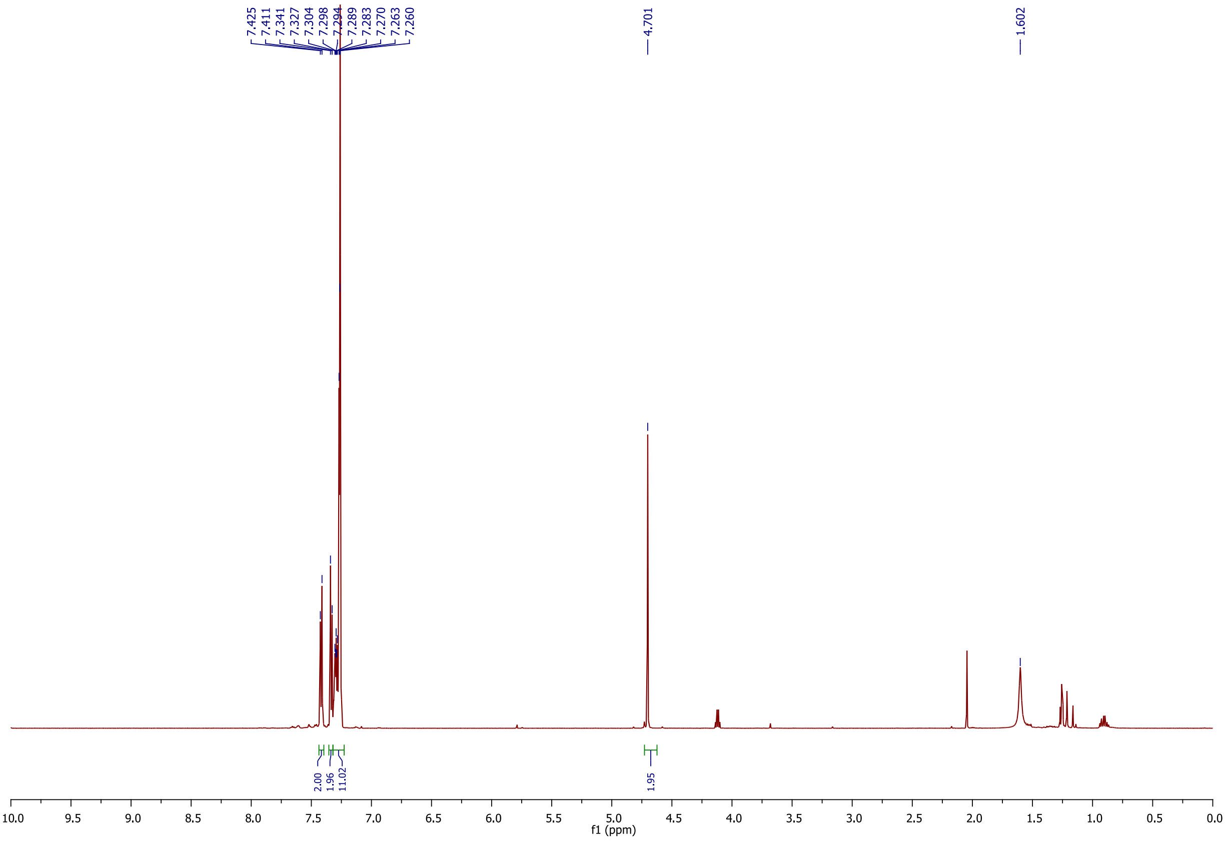
7.425
7.411
7.341
7.327
7.304
7.298
7.294
7.289
7.283
7.270
7.263
7.260
4.701
1.602
2.00
1.96
11.02
1.95
10.0 9.5 9.0 8.5 8.0 7.5 7.0 6.5 6.0 5.5 5.0 4.5 4.0 3.5 3.0 2.5 2.0 1.5 1.0 0.5 0.0
f1 (ppm)

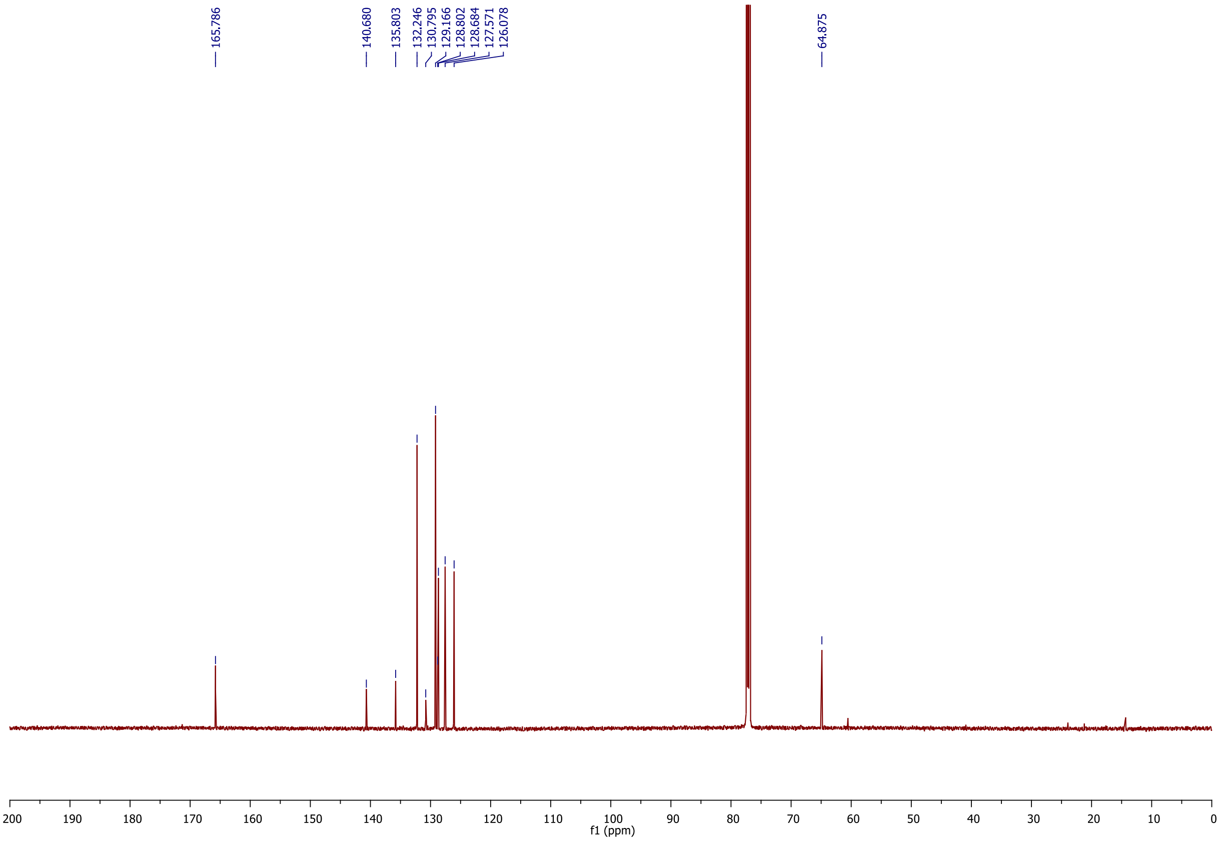
165.786
140.680
135.803
132.246
130.795
129.166
128.802
128.684
127.571
126.078
64.875
200 190 180 170 160 150 140 130 120 110 100 90 80 70 60 50 40 30 20 10 0
f1 (ppm)

This journal is © The Royal Society of Chemistry 2013

Carbonic acid 4-(2,5-dioxo-3,4-bis-phenylsulfanyl-2,5-dihydro-pyrrol-1-yl)-benzyl ester 4-nitro-phenyl ester 9

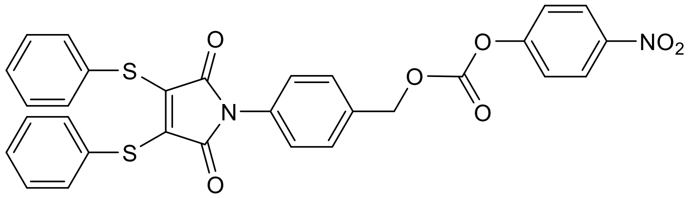

To a solution of 1-(4-hydroxymethyl-phenyl)-3,4-bis-phenylsulfanyl-pyrrole-2,5-dione **8** (30 mg, 0.071 mmol) in CH_2Cl_2 (2 mL) was added 4-nitrophenylchloroformate (17 mg, 0.085 mmol) and pyridine (0.007 mL, 0.085 mmol, and the reaction mixture was stirred at room temperature for 24 h. After this time, CH_2Cl_2 (10 mL) and 10% aqueous citric acid (10 mL) were added, the organic phase washed with H_2O (10 mL) and sat. NaCl (10 mL), dried ($MgSO_4$), filtered and the solvent removed in *vacuo*. The resultant crude residue was purified by flash column chromatography (20% EtOAc/petrol) to afford carbonic acid 4-(2,5-dioxo-3,4-bis-phenylsulfanyl-2,5-dihydro-pyrrol-1-yl)-benzyl ester 4-nitro-phenyl ester **9** as an oil (30 mg, 0.051 mmol, 72%): ^{1}H NMR ($CDCl_3$, 600 MHz) δ 8.27 (2H, d, J = 8.3 Hz, Ar$H$$NO_2$), 7.49 (2H, d, J = 8.3 Hz, Ar$H$$NO_2$), 7.40 (2H, d, J = 8.3 Hz, Ar$H$$CH_2O$), 7.37 (2H, d, J = 8.3 Hz, Ar$H$$CH_2O$), 7.33-7.26 (10H, m, Ar$H$S), 5.28 (2H, s, C$H_2$$OCO_2$); ^{13}C NMR ($CDCl_3$, 150 MHz) δ 165.6 (C), 155.5 (C), 152.5 (C), 147.6 (C), 135.9 (C), 133.8 (C), 132.3 (CH), 132.1 (C), 129.5 (CH), 129.2 (CH), 128.8 (CH), 128.7 (C), 126.2 (CH), 125.5 (CH), 121.9 (CH), 70.3 (CH_2); IR (neat) 3080, 1767, 1714, 1594, 1520 cm^{-1}; LRMS (EI) 584 (100, $[M]^+$); HRMS (EI) calcd for $C_{30}H_{20}O_7N_2S_2$ $[M]^+$ 584.0712, observed: 584.0718.

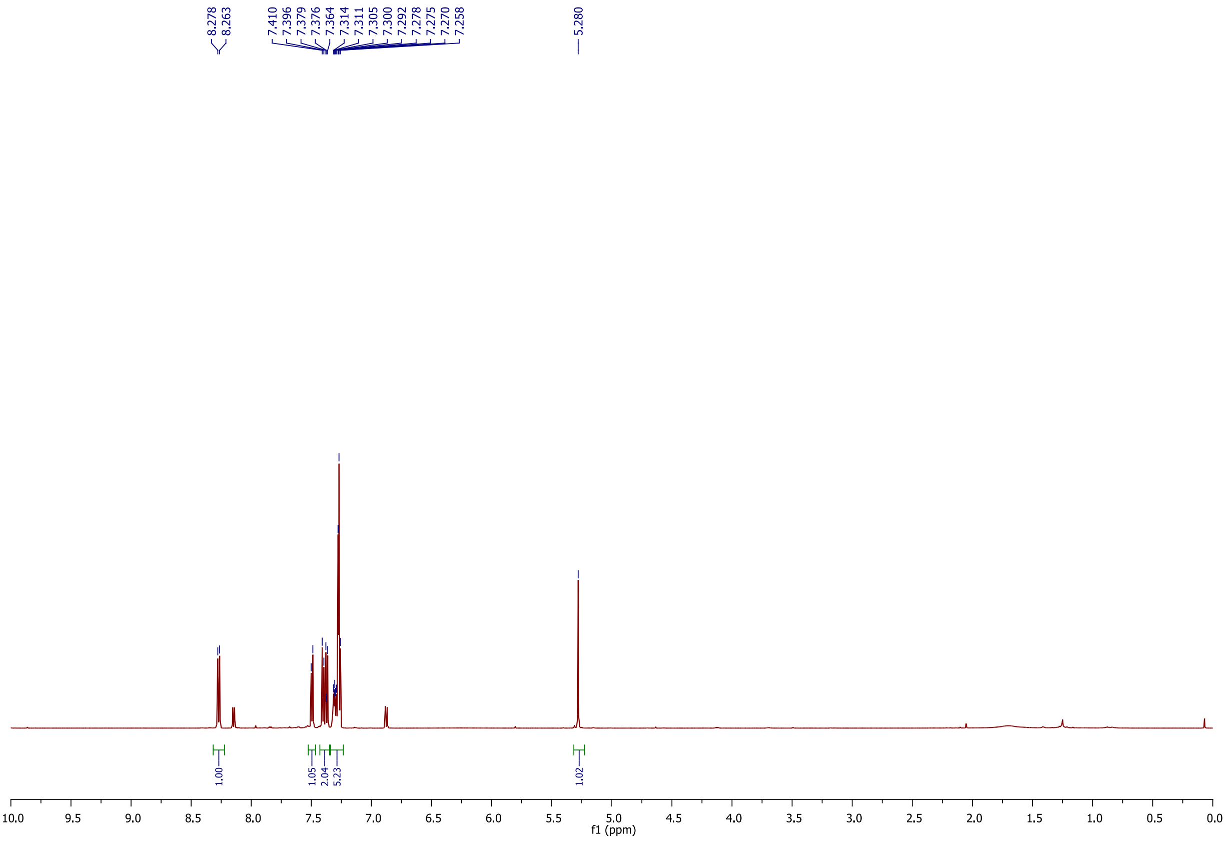

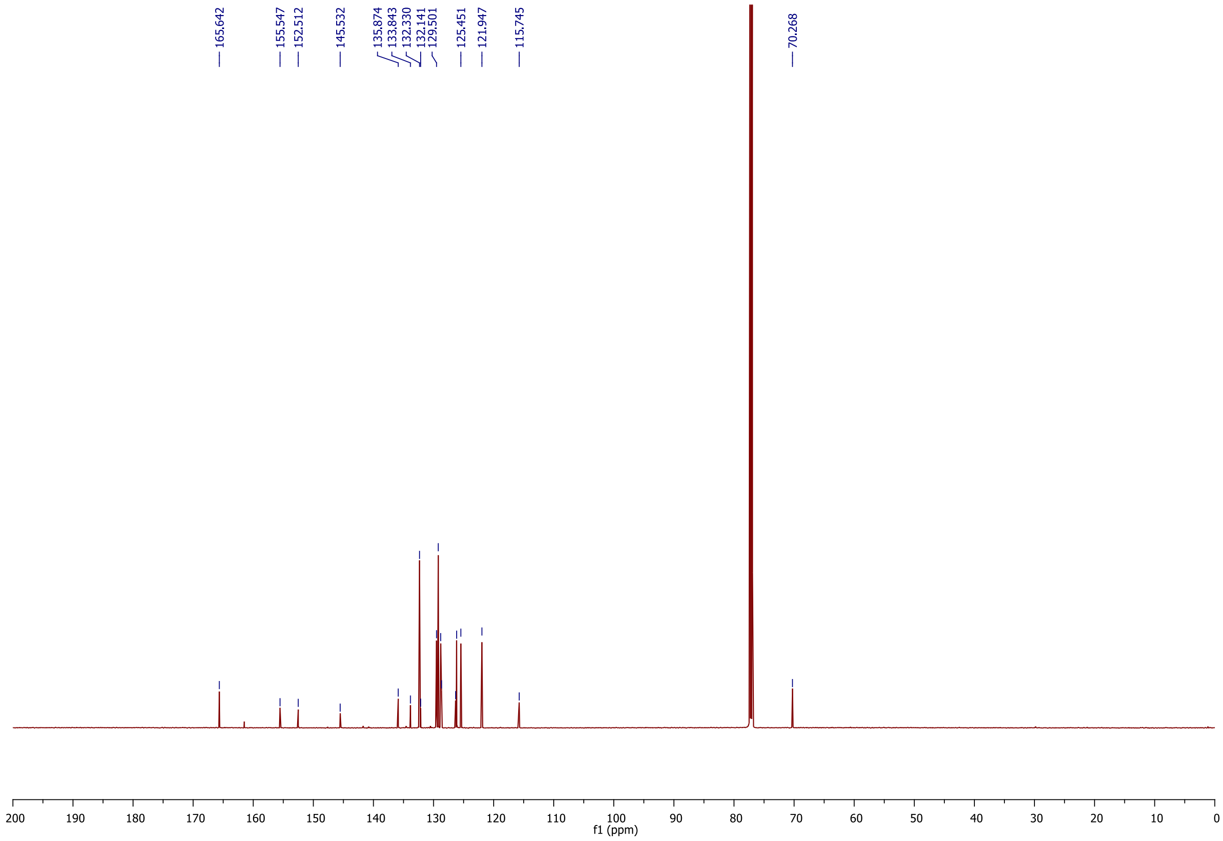

{3-Hydroxy-2-methyl-6-[3,5,12-trihydroxy-3-(2-hydroxy-acetyl)-10-methoxy-6,11-dioxo-1,2,3,4,6,11-hexahydro-naphthacen-1-yloxy]-tetrahydro-pyran-4-yl}-carbamic acid 4-(2,5-dioxo-3,4-bis-phenylsulfanyl-2,5-dihydro-pyrrol-1-yl)-benzyl ester 10

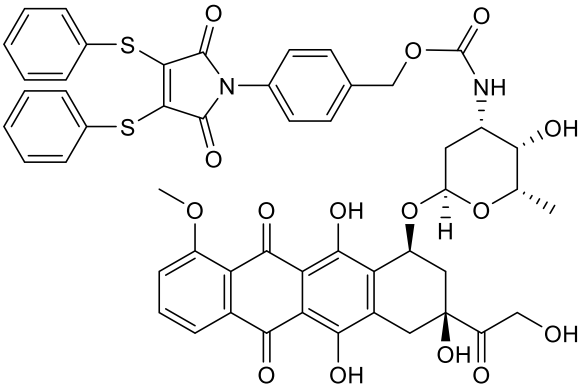

To a solution of carbonic acid 4-(2,5-dioxo-3,4-bis-phenylsulfanyl-2,5-dihydro-pyrrol-1-yl)-benzyl ester 4-nitro-phenyl ester **9** (9.8 mg, 0.017 mmol) and DOX·HCl (10 mg, 0.018 mmol) in NMP (0.30 mL) was added NEt_3 (2.5 µL, 0.018 mmol), and the reaction mixture stirred at room temperature for 3 days. After this time, the reaction mixture was diluted with 10% *i*-propanol/EtOAc, washed with H_2O (10 mL) and sat. NaCl (10 mL), dried ($MgSO_4$), filtered and the solvent removed in *vacuo*. The crude residue was purified by flash column chromatography (2% MeOH/CH_2Cl_2) to afford {3-Hydrohxy-2-methyl-6-[3,5,12-trihydroxy-3-(2-hydroxy-acetyl)-10-methoxy-6,11-dioxo-1,2,3,4,6,11-hexahydro-naphthacen-1-yloxy]-tetrahydro-pyran-4-yl}-carbamic acid 4-(2,5-dioxo-3,4-bis-phenylsulfanyl-2,5-dihydro-pyrrol-1-yl)-benzyl ester **10** as an orange oil (16.4 mg, 0.0166 mmol, 99%): ^{1}H NMR ($CDCl_3$, 600 MHz) δ 13.96 (1H, s , DOX), 13.22 (1H, s, DOX), 8.02 (1H, d, *J* = 8.2 Hz, DOX), 7.77 (1H, d, *J* = 8.2 Hz), 7.38 (1H, d, *J* = 8.2 Hz, DOX), 7.34 (2H, d, *J* = 8.2 Hz, Ar*H*CH_2), 7.31-7.22 (12H,

This journal is © The Royal Society of Chemistry 2013

m, Ar*H*CH_2 (2H) and Ar*H*S (10H)), 5.49 (1H, br s, DOX), 5.27 (1H, br s, DOX), 5.03-4.98 (2H, m, DOX), 4.75 (2H, s, CH_2O), 4.14-4.10 (1H, m, DOX), 4.07 (3H, s, DOX), 3.86-3.82 (1H, m, DOX), 3.65 (1H, s, DOX), 3.25 (1H, d, *J* = 18.7 Hz, DOX), 2.98 (1H, d, *J* = 18.7 Hz), 2.32 (1H, d, *J* = 14.6 Hz, DOX), 2.16 (1H, dd, *J* = 14.6 and 4.0 Hz, DOX), 1.86 (1H, dd, *J* = 13.4 and 4.9 Hz, DOX), 1.79-1.76 (1H, m, DOX), 1.27 (3H, d, *J* = 6.6 Hz, DOX); ^{13}C NMR ($CDCl_3$, 150 MHz) δ 214.0 (C), 187.2 (C), 186.8 (C), 165.7 (C), 161.1 (C), 156.3 (C), 155.7 (C), 155.5 (C), 136.1 (C), 135.9 (C), 135.7 (CH), 135.6 (C), 133.7 (C), 132.2 (CH), 131.2 (C), 129.2 (CH), 129.2 (CH), 128.9 (CH), 128.9 (C), 128.7 (CH), 125.9 (CH), 120.9 (C), 120.0 (CH), 118.6 (CH), 111.7 (C), 111.5 (C), 100.8 (CH), 76.7 (C), 69.7 (CH), 69.6 (CH), 67.4 (CH), 66.1 (CH_2), 65.7 (CH_2), 56.8 (CH_3), 47.1 (CH), 35.7 (CH_2), 34.1 (CH_2), 30.2 (CH_2), 17.0 (CH_3); IR (neat) 3481, 3080, 1716, 1580, 1517 cm^{-1}; LRMS (ESI) 1011 (100, $[M+Na]^+$); HRMS (ESI) calcd for $C_{51}H_{44}N_2O_{15}NaS_2$ $[M+Na]^+$ 1011.2081, observed: 1011.2122; $[\alpha]_D^{20}$ = +91.3 (*c* 1.1, $CHCl_3$).

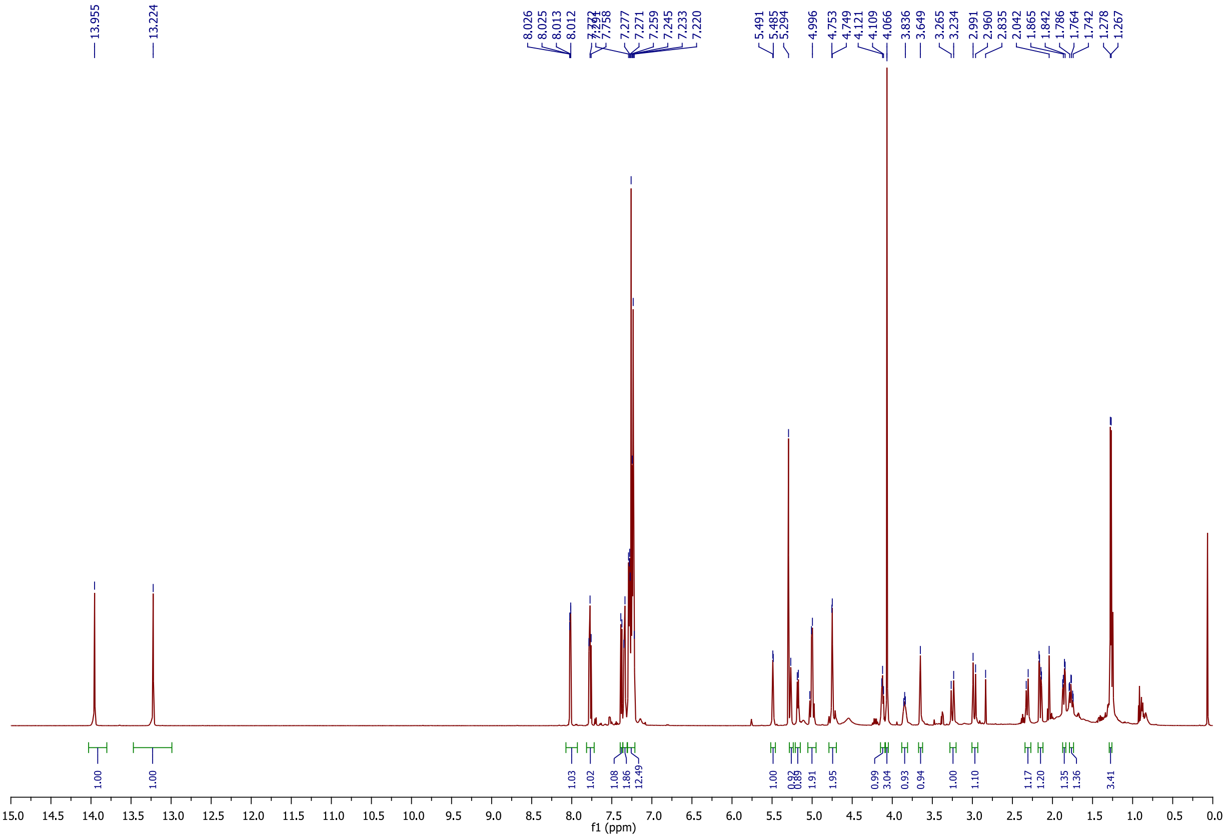

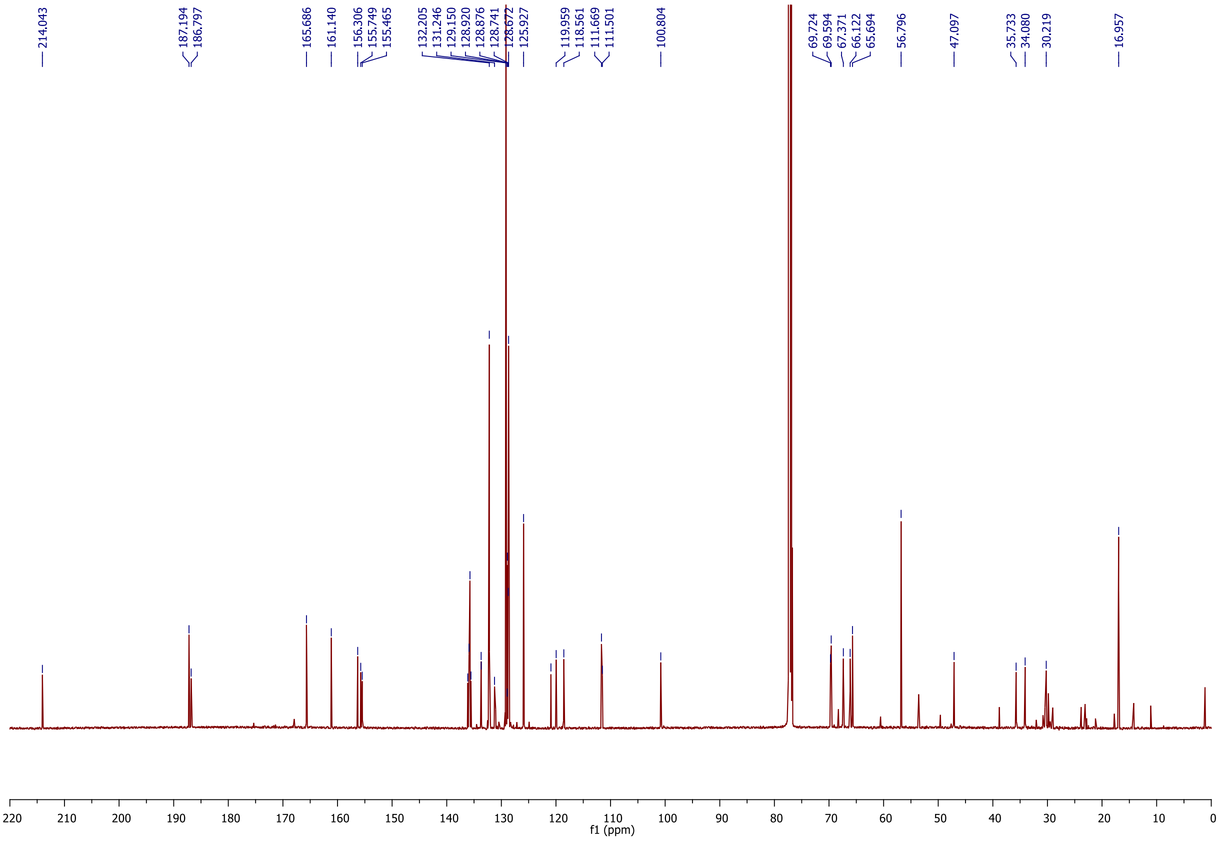
214.043
187.194
186.797
165.686
161.140
156.306
155.749
155.465
132.205
131.246
129.150
128.920
128.876
128.741
128.672
125.927
119.959
118.561
111.669
111.501
100.804
69.724
69.594
67.371
66.122
65.694
56.796
47.097
35.733
34.080
30.219
16.957
220
210
200
190
180
170
160
150
140
130
120
110
100
90
80
70
60
50
40
30
20
10
0
f1 (ppm)

This journal is © The Royal Society of Chemistry 2013

Trastuzumab Fab Fragment Preparation

Attempted preparation of a Trastuzumab Fab Fragment using the protocol outlined by K. L. Bennett *et al.*[3]

In accordance with the report by K. L. Bennett *et al.*,[3] trastuzumab (6.41 mg/ml) in digestion buffer was reacted with a 1/10 amount (wt./wt.) of immobilized papain (250 μg/mL of gel) for 20 h at 37 °C under nitrogen in a buffer containing 20 mM sodium phosphate monobasic, 10 mM disodium EDTA and 80 mM cysteine.HCl (pH 7.0). The cysteine.HCl was incorporated immediately before trastuzumab digestion. After digestion, the mixture was centrifuged at 200 rcf for 5 min and the supernatant was removed for purification. The supernatant was concentrated to a volume of 200 μL using a diafiltration column (30 KDa MWCO) to purify it from low-molecular-weight proteolytic contaminants, and buffer exchanged into phosphate-buffered saline (PBS, pH 7.0) by passage through diafiltration columns (30 KDa MWCO) four times with excessive PBS (pH 7.0). Finally, the sample was analysed by LCMS and revealed a mixture of Fab products, LCMS observed masses: 47306 and 47673.

(a)

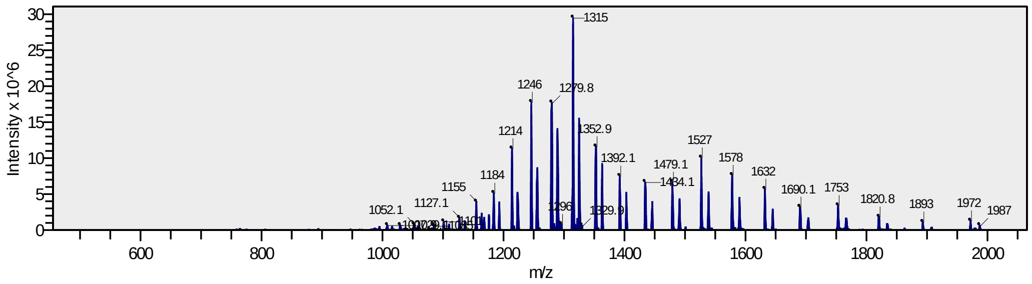

(b)

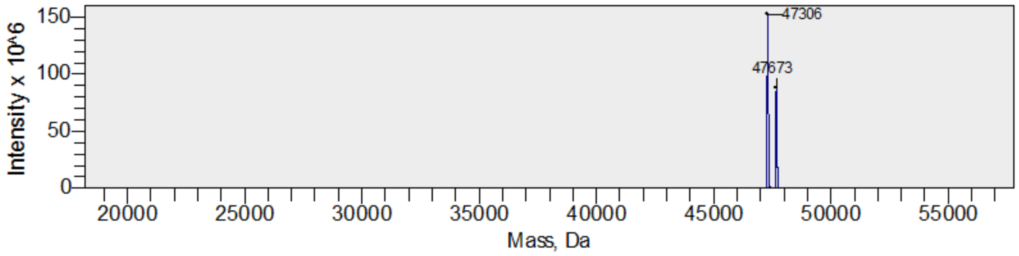

Figure S1. (a) non-deconvoluted and (b) deconvoluted MS data for attempted preparation of a trastuzumab Fab fragment using the protocol outlined by K. L. Bennett *et al.*[3]

.

Preparation of trastuzumab Fab fragment (11) using sequential digests with pepsin and papain

Immobilized pepsin (0.15 mL) was washed with digestion buffer (20 mM sodium acetate trihydrate, pH 3.1) four times and trastuzumab (0.5 mL, 6.41 mg/mL in digestion buffer) was added. The mixture was incubated for 5 h at 37 ºC whilst shaking (1100 rpm). The resin was separated from the digest using a filter column, and washed with digest buffer (50 mM phosphate, 1 mM EDTA, 150 mM NaCl, pH 6.8) three times. The digest was combined with the washes and the volume adjusted to 0.5 mL. The sample was analysed by LCMS and revealed formation of trastuzumab-F(ab')$_2$, LCMS observed mass: 97303.

This journal is © The Royal Society of Chemistry 2013

(a)

(b)

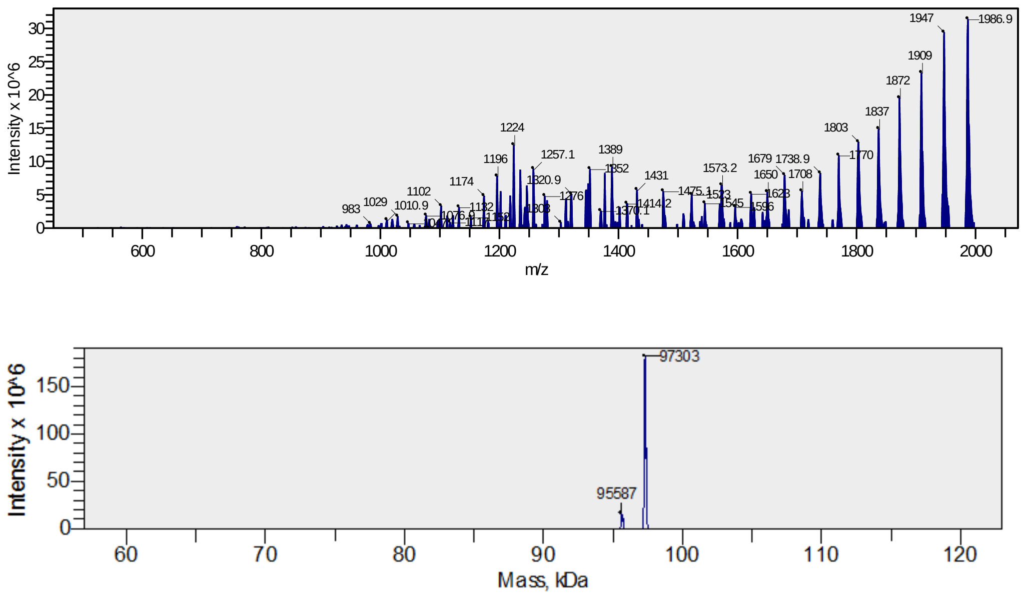

Figure S2. (a) non-deconvoluted and (b) deconvoluted MS data for digestion of trastuzumab with pepsin to afford trastuzumab-F(ab')$_2$.

After this, papain (1 mL, 0.25 mg/mL) was activated with 10 mM DTT (in digest buffer: 50 mM phosphate, 1 mM EDTA, 150 mM NaCl, pH 6.8) under an argon atmosphere whilst shaking (1100 rpm) for 1 h at 25 ºC in the dark. The resin was washed with digest buffer (without DTT) four times and the 0.5 mL of Herceptin-F(ab')$_2$ added. The mixture was incubated for 16 h at 37 ºC whilst shaking (1100 rpm) in the dark. Then the resin was separated from the digest using a filter column, and washed with PBS (pH 7.0) three times. The digest was combined with the washes and the buffer was exchanged completely for PBS (pH 7.4) using diafiltration columns (10 KDa MWCO) and the volume adjusted to 0.4 mL. The digest was analysed by SDS-PAGE and LCMS to reveal formation of a single trastuzumab Fab fragment, **11**: observed mass 47644. The concentration of trastuzumab Fab fragment **11** was determined by UV/VIS using a molecular extinction coefficient of ε_{280} = 68590 M^{-1} cm^{-1}. [trastuzumab Fab fragment **11**] 3.33 mg/mL (0.4 mL), 64%.

(a)

(b)

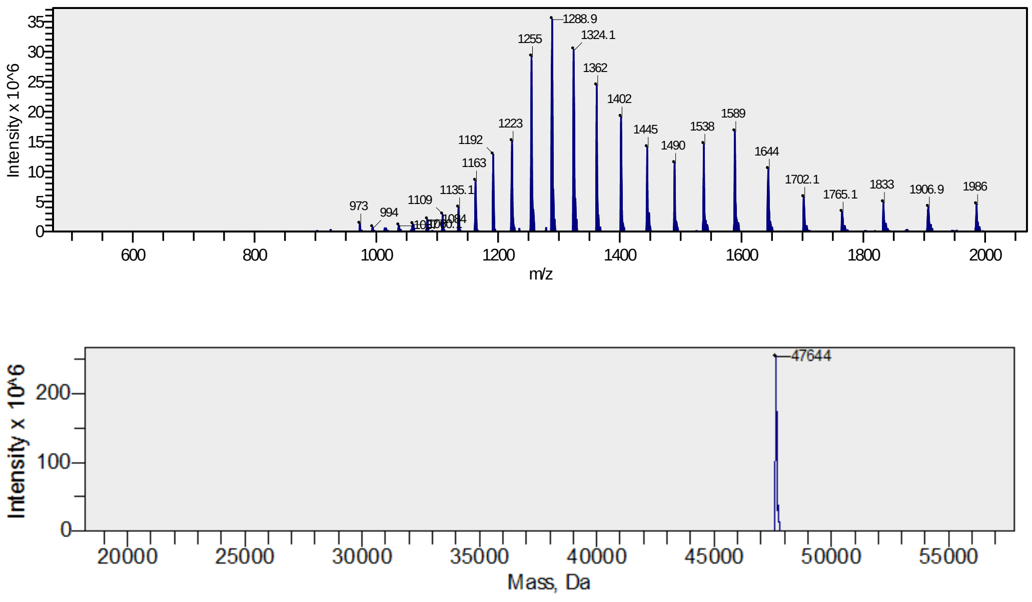

This journal is © The Royal Society of Chemistry 2013

(c)

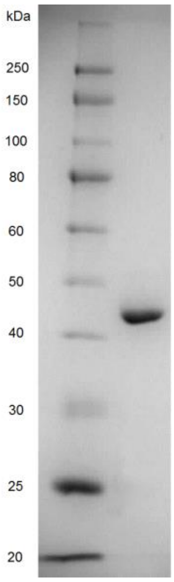

Figure S3. (a) non-deconvoluted, (b) deconvoluted MS data, and (c) SDS-PAGE characterisation of trastuzumab Fab fragment **11** prepared by using sequential digests with pepsin and papain.

Fab ADC Experimental Procedures and Data

Reduced trastuzumab Fab

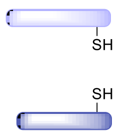

To a solution of Fab fragment **11** (50 μL, 1.72 mg/mL, 25 mM sodium borate, 25 mM NaCl, 1 mM EDTA, pH 8.0) was added TCEP (15 μL, 0.36 mM) to affect reduction of the interchain disulfide. After 1.5 h at 37 °C, the reaction mixture was analysed by LCMS to reveal the heavy and light chains only (*i.e.* reduced trastuzumab Fab fragment).

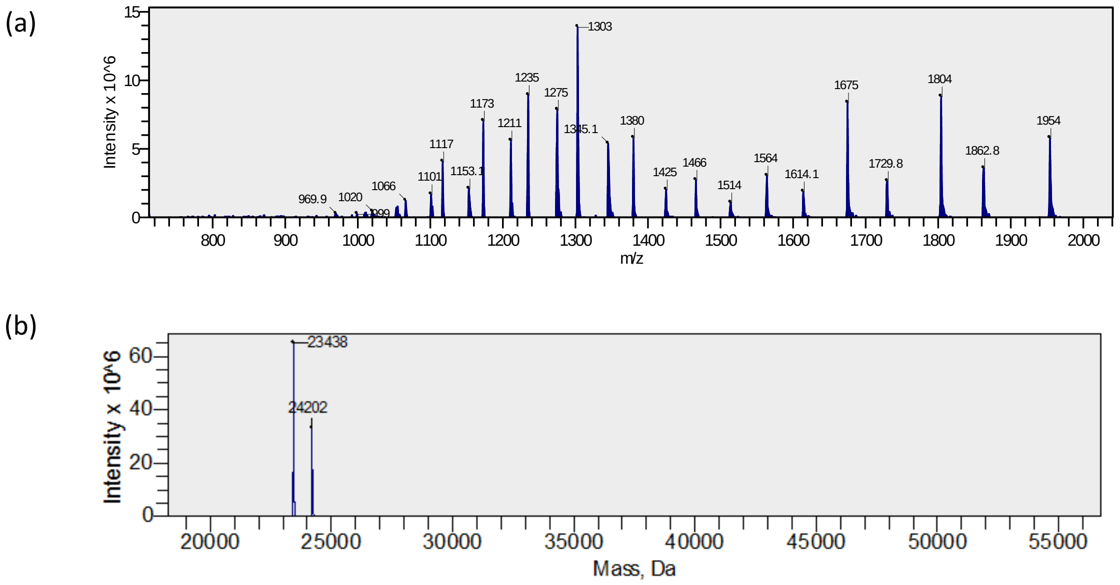

Figure S4. (a) non-deconvoluted and (b) deconvoluted MS data for reduction trastuzumab Fab fragment **11** with TCEP.

Trastuzumab Fab-DOX ADC 12

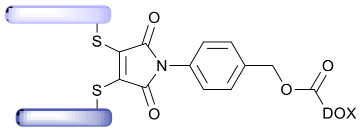

To the solution of reduced trastuzumab Fab fragment **11** was added MeCN (13 μL, 20% v/v), DMF (6.5 μL, 10% v/v) and {3-Hydroxy-2-methyl-6-[3,5,12-trihydroxy-3-(2-hydroxy-acetyl)-10-methoxy-6,11-dioxo-1,2,3,4,6,11-hexahydro-naphthacen-1-yloxy]-tetrahydro-pyran-4-yl}-carbamic acid 4-(2,5-dioxo-3,4-bis-phenylsulfanyl-2,5-dihydro-pyrrol-1-yl)-benzyl ester **10** (1.15 μL, 7.91 mM in DMF), and the reaction maintained at 37 °C for 1 h. After this time, the reaction mixture was analysed by LCMS to reveal formation of desired trastuzumab Fab-DOX ADC **12** (expected mass: 48414 Da, observed mass: 48413 Da).

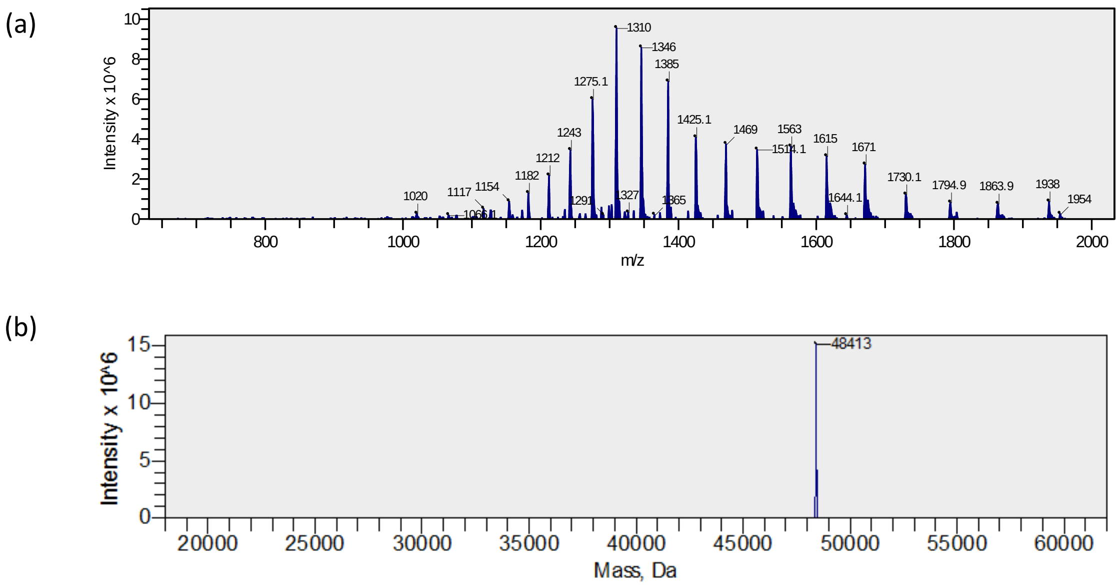

Figure S5. (a) non-deconvoluted and (b) deconvoluted MS data for trastuzumab Fab-DOX ADC **12**.

Trastuzumab Fab-DOX ADC 13

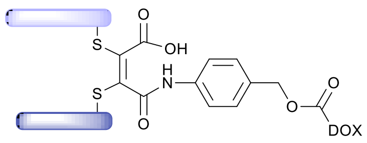

The solution of trastuzumab Fab-DOX ADC **12** was prepared as described above, and then buffer exchanged into PBS (pH 7.4, final volume: 50 µL), and the solution incubated for 20 h at 37 °C. After this time, the reaction mixture was analysed by LCMS to reveal formation of partial maleimide hydrolysis product trastuzumab Fab-DOX ADC **13** (expected mass: 48432 Da, observed mass: 48433 Da). The only degradation product (expected mass: 48036 Da, observed mass: 48031 Da) observed corresponded to that resulting from the known hydrolysis of the sugar component of DOX upon prolonged incubation.[4]

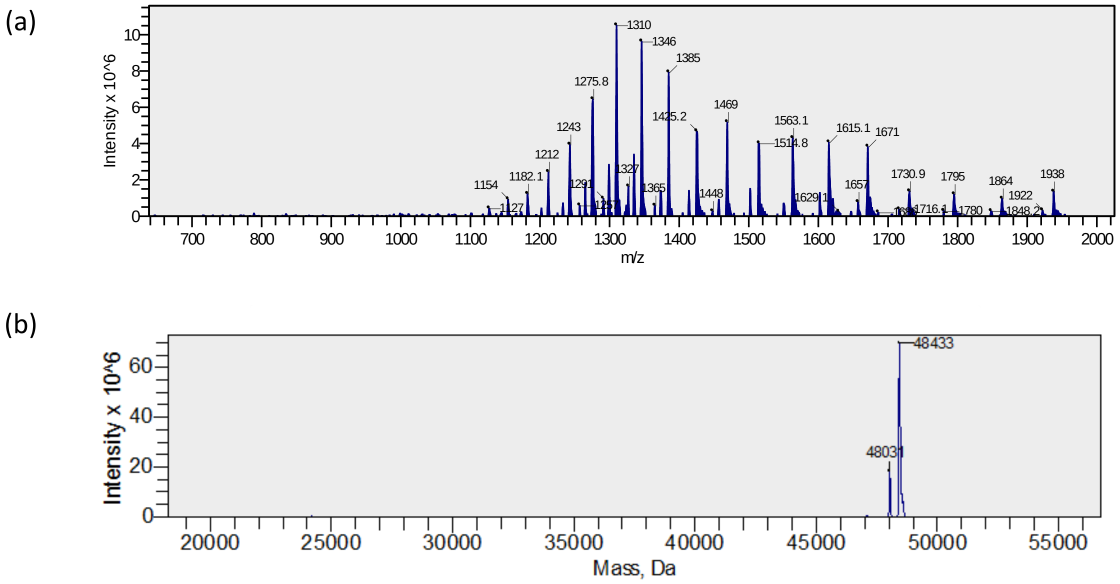

Figure S6. (a) non-deconvoluted and (b) deconvoluted MS data for trastuzumab Fab-DOX ADC **13**.

Stability of trastuzumab Fab-DOX ADC (13) at physiological pH and temperature

Trastuzumab Fab-DOX ADC **13** was incubated at physiological temperature and pH (PBS, pH 7.4, final volume: 50 µL) for 3 days. During this time, no degradation of the linker was observed by LCMS.

(a)

(b)

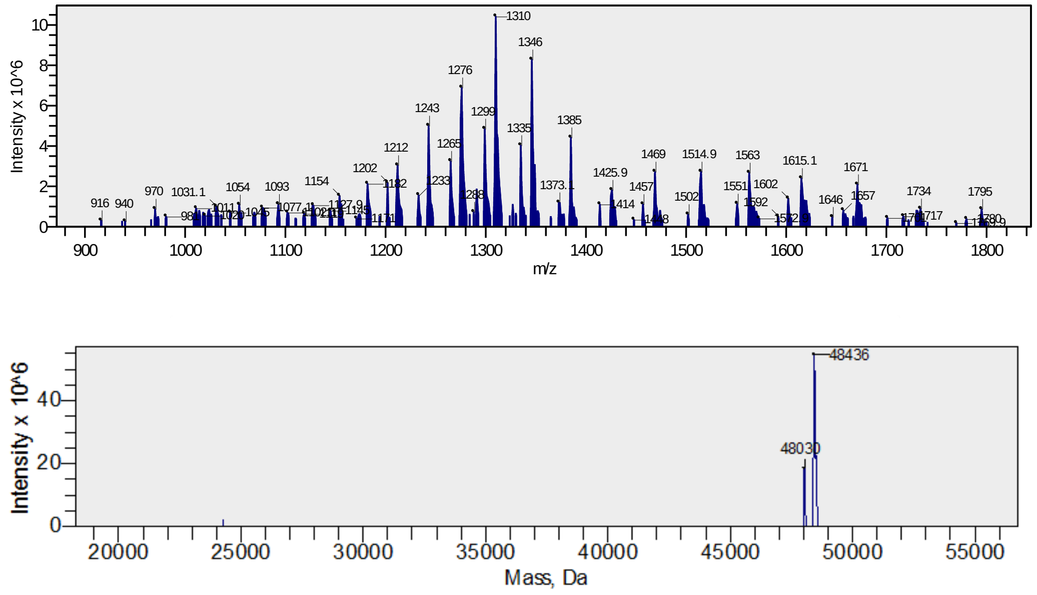

Figure S7. (a) non-deconvoluted and (b) deconvoluted MS data for trastuzumab Fab-DOX ADC **13** at physiological pH and temperature after 3 days.

Release of DOX from ADC 13 at lysosomal pH to yield Conjugate 14

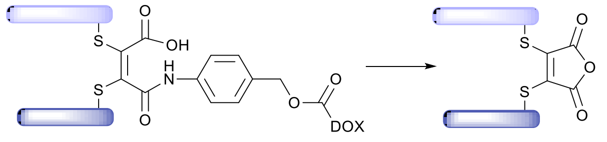

The solution of trastuzumab Fab-DOX ADC **13** was prepared as described above and then buffer exchanged into a low pH buffer (50 mM citric acid, 150 mM sodium chloride, pH 4.5) by ultrafiltration. The solution was incubated at 37 ºC. Aliquots of the reaction mixture were analysed by LCMS after 2, 6, 24, 48 and 72 h. Near quantitative release of the cargo was observed (expected mass: 47740 Da, observed mass: 47740 Da).

(a)

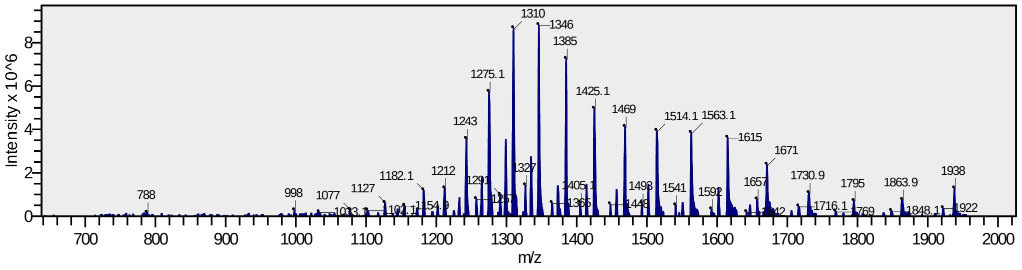

2h

(b)

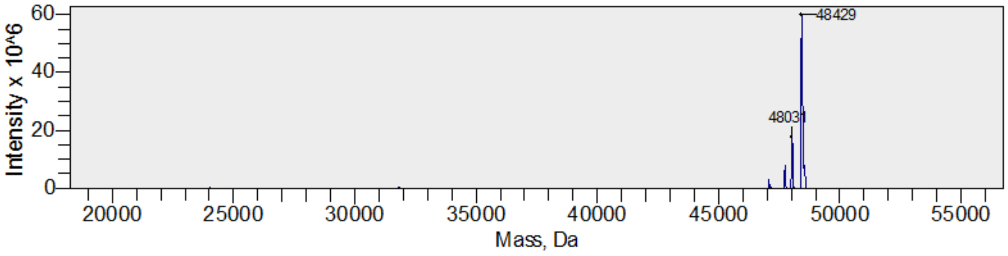

2h

Figure S8. (a) non-deconvoluted and (b) deconvoluted MS data for trastuzumab Fab-DOX ADC **13** at pH 4.5 and 37 ºC after 2 h.

(a)

(b)

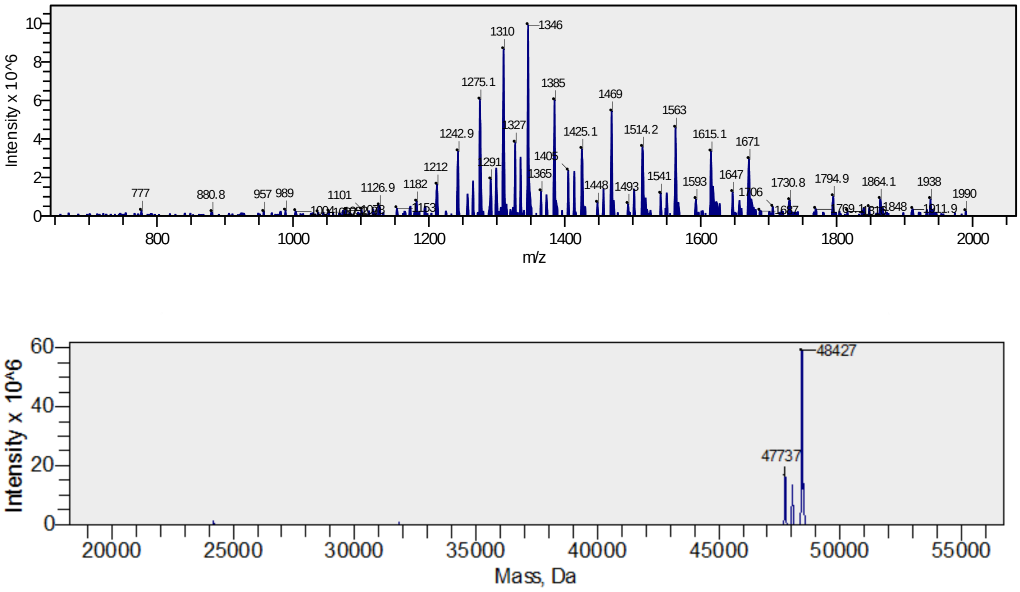

6h

6h

Figure S9. (a) non-deconvoluted and (b) deconvoluted MS data for trastuzumab Fab-DOX ADC **13** at pH 4.5 and 37 ºC after 6 h.

(a)

(b)

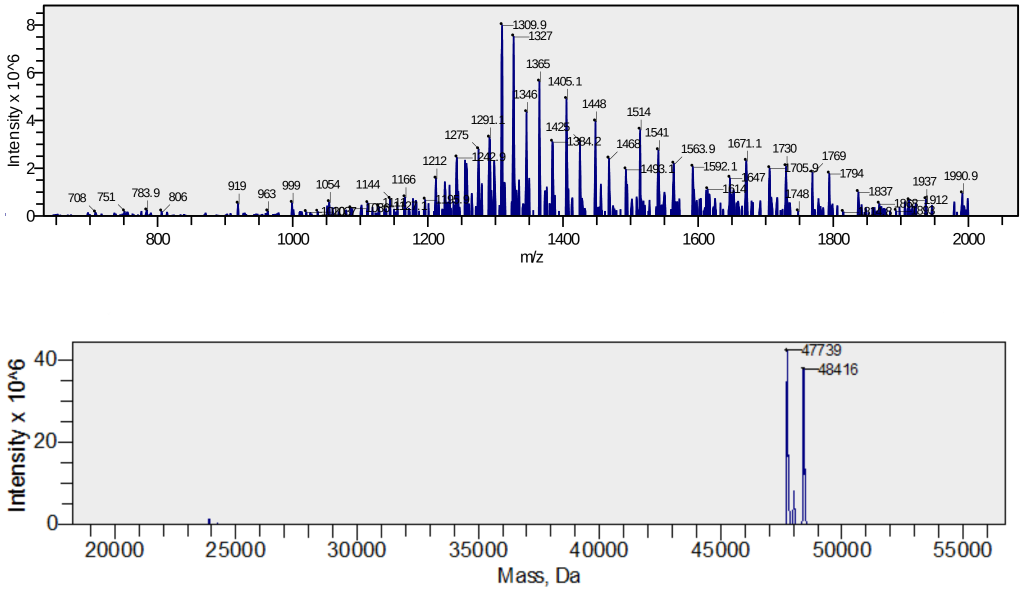

24h

24h

Figure S10. (a) non-deconvoluted and (b) deconvoluted MS data for trastuzumab Fab-DOX ADC **13** at pH 4.5 and 37 ºC after 24 h.

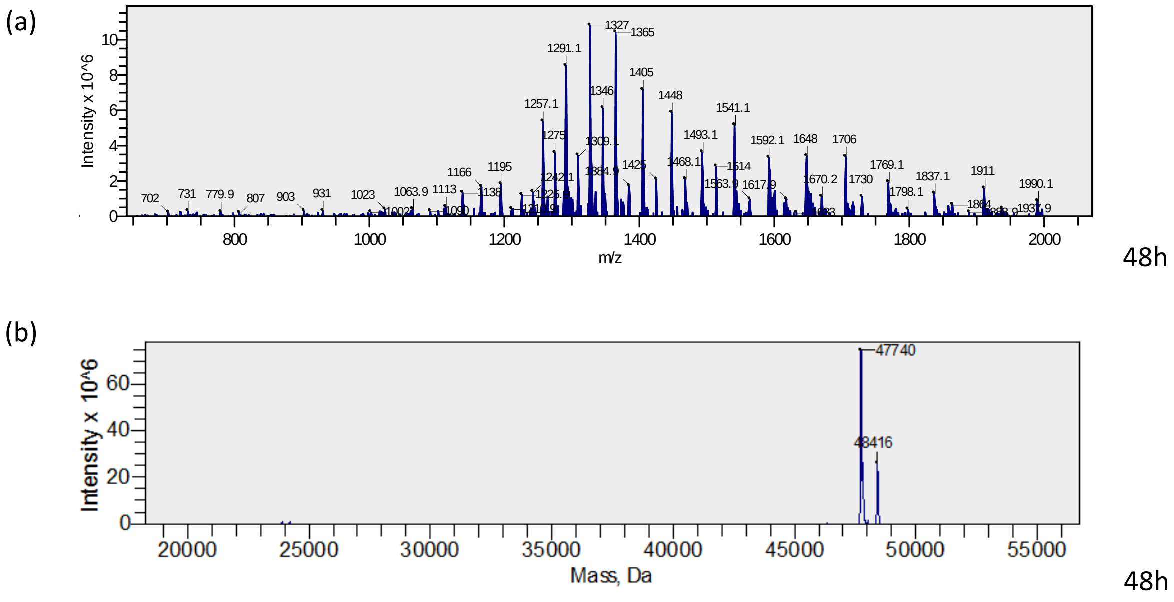

Figure S11. (a) non-deconvoluted and (b) deconvoluted MS data for trastuzumab Fab-DOX ADC **13** at pH 4.5 and 37 °C after 48 h.

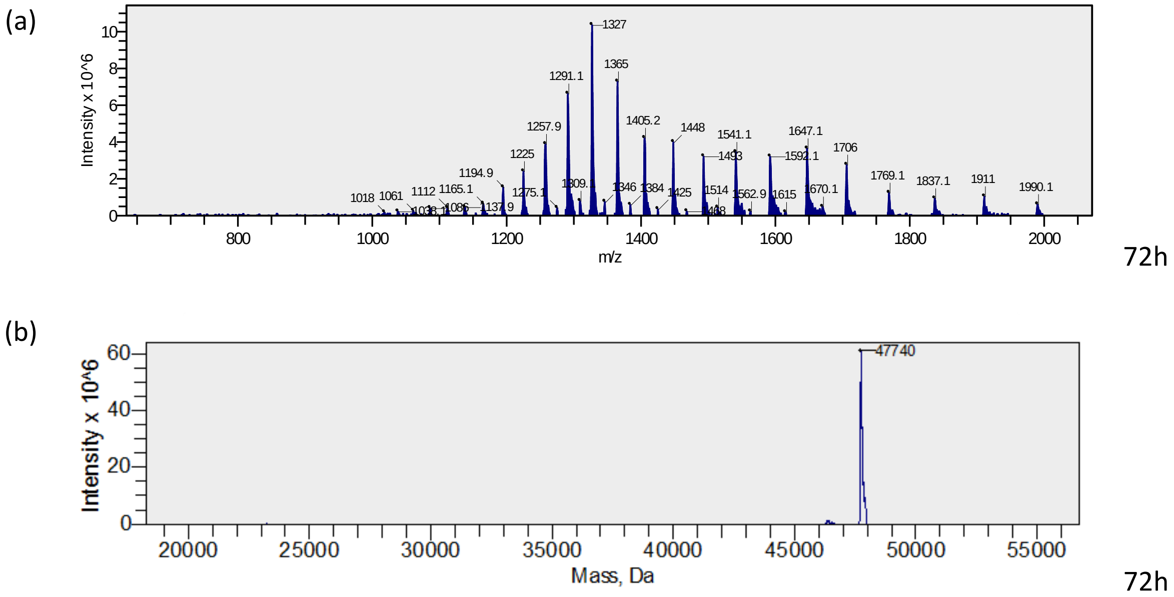

Figure S12. (a) non-deconvoluted and (b) deconvoluted MS data for trastuzumab Fab-DOX ADC **13** at pH 4.5 and 37 °C after 72 h.

Preparation of processed trastuzumab Fab

A solution of Fab fragment **11** (50 μL, 1.72 mg/mL, 25 mM sodium borate, 25 mM NaCl, 1 mM EDTA, pH 8.0) was incubated for 1.5 h at 37 °C. After this time, to the solution were added MeCN (13 μL, 20% v/v) and DMF (6.5 μL, 10% v/v) and the reaction maintained at 37 °C for 1 h. Next, the reaction mixture was buffer exchanged into PBS (pH 7.4, final volume: 50 μL), and the solution incubated for 20 h at 37 °C. After this time, the reaction mixture was analysed by LCMS to reveal processed Fab (expected mass: 47644 Da, observed mass: 47650 Da).

This journal is © The Royal Society of Chemistry 2013

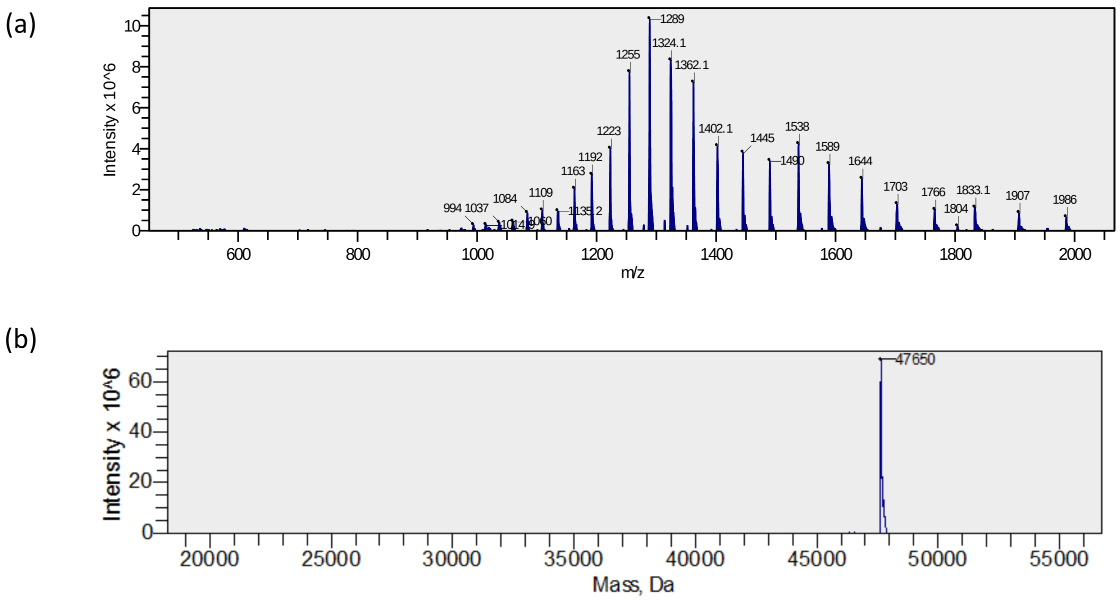

Figure S13. (a) non-deconvoluted and (b) deconvoluted MS data for processed trastuzumab Fab.

Protocol for ELISA

96-Well plates were coated overnight at 4 °C with HER2 (0.25 µg/mL); PBS was used as negative control. HER2 was removed; the wells washed with PBS and blocked with 200 µL of 1% BSA solution for 2 h at room temperature. After washing with PBS, the serially diluted test samples (30 nM, 10 nM, 3.33 nM, 1.11 nM, 0.37 nM, 0.12 nM) were added and incubated for 1 h at room temperature. The wells were washed twice with PBS-T (PBS, 0.1% Tween-20) and once with PBS, and anti-human IgG, Fab-specific-HRP antibody (Sigma-Aldrich) was incubated for 1 h at room temperature to detect the bound test sample. The plates were washed again and 100 µL of 0.5 mg/mL *o*-phenylenediamine hydrochloride (Sigma-Aldrich) in a phosphate-citrate buffer with sodium perborate were added as substrate. Once colour was visible, the reaction was stopped by acidifying with 50 µL of 4M HCl. Absorbance was immediately measured at 490 nm. ELISA assays were conducted for trastuzumab Fab **11**, processed Fab and Fab ADC **13**.

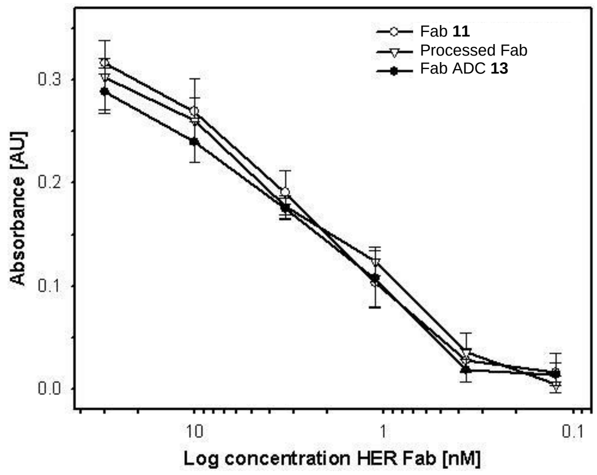

Figure S14. 1 ELISA analysis of Fab **11**, processed Fab and Fab ADC **13** binding to the HER2 antigen.

References

1. M. Dubernet, V. Caubert, J. Guillard, M.-C. Viaud-Massuard, *Tetrahedron*, 2005, **61**, 4585.

This journal is © The Royal Society of Chemistry 2013

2. L. Castañeda, Z. V. F. Wright, C. Marculescu, T. M. Tran, V. Chudasama, A. Maruani, E. A. Hull, J. P. M. Nunes, R. J. Fitzmaurice, M. E. B. Smith, L. H. Jones, S. Caddick, J. R. Baker, *Tetrahedron Lett.*, 2013, **54**, 3493.

3. K. L. Bennett, S. V. Smith, R. J. W. Truscott, M. M. Sheil, *Anal. Biochem.*, 1997, **245**, 17.

4. L. A. Trissel, in *Handbook on Injectable Drugs*, Ed. American Society of Health-System Pharmacists, MacMillan Press, Basingstoke, 9th edn., 1996, 379.
